# Biogeography of Mediterranean Hotspot Biodiversity: Re-Evaluating the 'Tertiary Relict' Hypothesis of Macaronesian Laurel Forests

**DOI:** 10.1371/journal.pone.0132091

**Published:** 2015-07-14

**Authors:** Paulina Kondraskov, Nicole Schütz, Christina Schüßler, Miguel Menezes de Sequeira, Arnoldo Santos Guerra, Juli Caujapé-Castells, Ruth Jaén-Molina, Águedo Marrero-Rodríguez, Marcus A. Koch, Peter Linder, Johanna Kovar-Eder, Mike Thiv

**Affiliations:** 1 Botany Department, State Museum of Natural History Stuttgart, Stuttgart, Germany; 2 Universidade da Madeira, Centro de Ciências da Vida, Funchal, Madeira, Portugal; 3 Unidad de Botánico (ICIA), Puerto de la Cruz, Tenerife, Spain; 4 Jardin Botanico Canario "Viera y Clavijo"-Unidad Asociada CSIC, Las Palmas de Gran Canaria, Spain; 5 Dept. Biodiversity und Plant Systematics, University of Heidelberg, Heidelberg, Germany; 6 Institute of Systematic Botany, University of Zurich, Zurich, Switzerland; Royal Botanic Gardens, Kew, UNITED KINGDOM

## Abstract

The Macaronesian laurel forests (MLF) are dominated by trees with a laurophyll habit comparable to evergreen humid forests which were scattered across Europe and the Mediterranean in the Paleogene and Neogene. Therefore, MLF are traditionally regarded as an old, 'Tertiary relict' vegetation type. Here we address the question if key taxa of the MLF are relictual. We evaluated the relict hypothesis consulting fossil data and analyses based on molecular phylogenies of 18 representative species. For molecular dating we used the program BEAST, for ancestral trait reconstructions BayesTraits and Lagrange to infer ancestral areas. Our molecular dating showed that the origins of four species date back to the Upper Miocene while 14 originated in the Plio-Pleistocene. This coincides with the decline of fossil laurophyllous elements in Europe since the middle Miocene. Ancestral trait and area reconstructions indicate that MLF evolved partly from pre-adapted taxa from the Mediterranean, Macaronesia and the tropics. According to the fossil record laurophyllous taxa existed in Macaronesia since the Plio- and Pleistocene. MLF are composed of species with a heterogeneous origin. The taxa dated to the Pleistocene are likely not 'Tertiary relicts'. Some species may be interpreted as relictual. In this case, the establishment of most species in the Plio-Pleistocene suggests that there was a massive species turnover before this time. Alternatively, MLF were largely newly assembled through global recruitment rather than surviving as relicts of a once more widespread vegetation. This process may have possibly been triggered by the intensification of the trade winds at the end of the Pliocene as indicated by proxy data.

## Introduction

**Fig 1:**
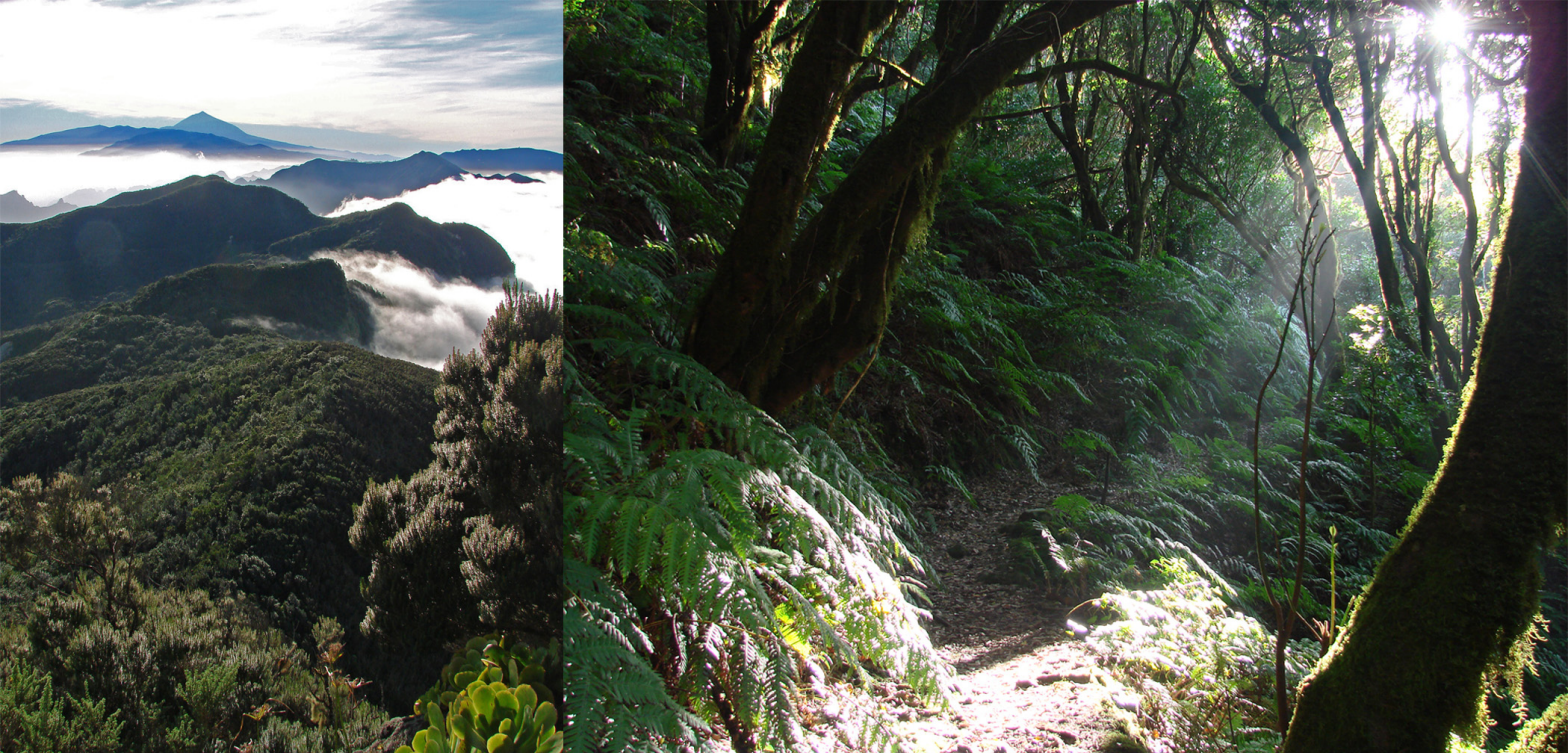
Laurel forest in the Anaga Mountains on Tenerife (photos A. Betzin).

Biodiversity hotspots are particularly important for conservation strategies [1]. Besides spatial patterns and levels of biodiversity, biotas with high species numbers also have a phylogenetic and a temporal dimension. The evolution of species assemblies is, however, poorly studied. Temporal patterns have been proposed for Macaronesian laurel forests (MLF). Traditionally, they are interpreted as an old biome and as a typical example of a ‘Tertiary (65–2.6 Ma) relict’ vegetation type [2, 3] (Fig 1; S1 Table). They occur on the Canary Islands, Madeira and Azores and are part of the Mediterranean biodiversity hotspot [4].

The Macaronesian archipelagos constitute geological hotspot island series situated in the Atlantic Ocean [3]. Although the hotspots existed since the Palaeocene (66.0–56.0 Ma), the oldest extant island, Selvagem Pequena, dates back to ca. 29.5–28.7 Ma [5] and the youngest island (Pico, in the Azores) to ca. 0.300–0.037 Ma [6]. The climate of the islands was initially tropical [3] and has changed extensively during the Neogene (23.0–2.6 Ma) [7]. Analyses of sedimentological and geochemical proxies revealed changes in climate and trade wind systems during the glacials and interglacials [8], [9]. During the Pleistocene (2.6–0.01 Ma), the trade wind zone has likely moved over the islands. Currently, the windward mid slopes receive regular orographic moisture from the trade winds and are characterised by distinctive laurel forests. In contrast, the lower and leeward sides of the mountains harbour drought- and heat-adapted sclerophyllous or succulent vegetation [10].

Laurel forests are dominated by trees with evergreen, entire, elongated and glossy leaves. They are globally distributed in small patches with an aseasonal, humid and frost-free climate. On the Macaronesian islands, laurel forests usually occur on the north-eastern (windward) slopes at 600–1200 m, where trade winds frequently deposit orographic rain and mist. Most of the constituent species of these forests are nowadays restricted to the Macaronesian islands, where they are relatively common. We recognised a total of 100 characteristic and non-characteristic genera occurring in the MLF (S2 Table). These forests have been typically regarded as relictual because some of their most characteristic species (e.g. *Laurus novocanariensis*, *L*. *azorica*, and *Ocotea foetens*) have laurophyllous leaves which are morphologically very similar to fossil leaves from the Eocene (56.0–33.9 Ma) to the Pliocene (5.3–2.6 Ma; references in S3 Table, e.g. [11]; [12]). Laurophyllous vegetation was widespread in large parts of Europe in the Paleogene (66.0–23.0 Ma) until the mid-Miocene climatic optimum (17–15 Ma). The subsequent climatic decrease in temperature and precipitation led to a decline of laurophyllous vegetation in Central Europe, and several of these taxa retreated to southern Europe. By the end of the late Miocene-early Pliocene (11.6–3.6 Ma) laurophyllous elements were mainly restricted to SW-Europe, Italy, Balkans and western Georgia (Colchis) [13]. In a progressive, non-linear process, they were largely replaced by deciduous, mesophytic forests in the northern parts and sclerophyllous vegetation in the South [14]. European laurel forests were almost entirely lost by the end of Pliocene (2.6 Ma) [15], [13].

In this study, we test if the MLF are remnants of these European ‘Tertiary laurel forests’. This hypothesis implies several predictions: first, the constituent lineages should have been recruited before their extinction by the end of the Pliocene. The second prediction is that dispersal to Macaronesia was from the Mediterranean/Tethyan laurel forests. The third expectation is that the most recent common ancestor (mrca) of the MLF species and its sister group should also have occupied laurel forests. Time-calibrated phylogenies, ancestral state and area reconstructions were performed for 18 typical MLF taxa to study whether these criteria were met. Complementary to the molecular analysis we reviewed the fossil record of laurophyllous taxa to see if this is in accordance with the phylogenetic data.

## Material and Methods

### Taxon sampling and molecular markers

We aimed at analysing representative taxa of MLF. They are not a homogeneous vegetation type, because MLF taxa show different distribution patterns across the Canaries, Madeira and Azores (S2 Table), and sometimes within an archipelago, and the species composition may differ according to various microclimates. Nonetheless, MLF can be defined as vegetation types dominated by laurophyllous trees. Therefore, we selected angiosperm taxa according to the following criteria: 1) the taxa are exclusively restricted to MLF (ca. 60% of the MLF species), 2) they have different distribution patterns across the archipelagos, 3) they consist of different life forms characteristic of these forests (trees, shrubs and herbs) and 4) several of them are often cited examples of ‘Tertiary relicts’ (S1 Table). The occurrence of the included Crassulaceae, *Aeonium cuneatum* and *Aichryson pachycaulon*, on rock habitats may not be typical for MLF. Lösch (1990) [16], however, showed that these taxa are eco-physiologically adapted to wet conditions of laurel forests.

For our question it is essential to include the closest relatives of the studied MLF taxa and suitable taxa for calibration. Accordingly, a wide range of taxonomic levels had to be considered. Our strategy was to include predominantly newly generated DNA sequences of MLF taxa into modified data sets of published phylogenies. We aimed at considering several accessions of MLF species to identify potential cases of taxonomic incongruence. The methods employed are sensitive to incomplete sampling. This could affect our analysis in different ways: 1) molecular dating might be biased in such a way that the resulting branch lengths of the MLF taxa would likely become shorter if a yet undetected sister group was added. Therefore, the actual age estimates may be younger than our estimated ones (error type II). Incomplete sampling may also have an effect on 2) ancestral trait/area reconstructions. If the sister group is missed in the analyses, the ancestral traits and areas could be wrongly inferred (error type I). For some species-rich genera like *Euphorbia* and *Ocotea* we focussed on the closest relatives of the corresponding clade including the MLF taxon or clade. For *Euphorbia* we could rely on an extensive published phylogeny (S5 Table). This was not the case for *Ocotea* and therefore, we could only sample very few of the ca. 350 species. Accordingly, we decided to consider *Ocotea foetens* only for molecular dating, possibly overestimating the age, and to omit this taxon from ancestral traits/areas reconstructions. We want to emphasise that our taxon sampling reflects the most recent knowledge on the phylogenies of the MLF taxa, but that this is still an ongoing effort. Our choice of DNA markers (S4 Table) depended on those used in the previous studies. Due to these limitations, for some taxa only single gene trees could be analysed. In many cases nrITS was used despite several known problems [17] providing phylogenetic hypotheses which may, however, differ from those inferred from others markers. For *Ixanthus* and *Bystropogon*, we combined datasets from different markers because no incongruences between chloroplast and nuclear markers were observed in the tree topology of the taxa of interest.

### DNA sequencing

DNA was extracted from silica dried samples or herbarium material using the DNeasy Plant Mini Kit (Qiagen) according to the manufacturer’s protocol. Amplification of nrITS was performed in a volume of 25 μl, containing 1× PCR reaction buffer, 2 mM MgCl_2_, 0.12 mM dNTPs, 0.05 U/μl DreamTaq DNA polymerase (Thermo Scientific), 0.28 μM primer (primers as used in previous publications, see S5 Table) and 5 ng/μl DNA template. Amplification of *mat*K was obtained with 1× PCR reaction buffer, 25 mM MgCl_2_, 0.12 mM dNTPs, 0.05 U/μl DreamTaq DNA polymerase, 0.28 μM primer and 5 ng/μl DNA template. Reactions were performed on a Biometra thermocycler under the following conditions: (nrITS:) 3 min 95°C, 9 cycles (45 s 95°C, 45 s 60°C, 90 s 72°C), 29 cycles (45 s 95°C, 45 s 55°C, 90 s 72°C), 10 min 72°C; (*mat*K:) 3 min 94°C, 9 cycles (20 s 94°C, 40 s 60°C (- 0,5°C per cycle), 90 s 72°C), 29 cycles (45 s 94°C, 30 s 50°C, 90 s 72°C), 10 min 72°C. PCR products were cleaned using a purification kit (Qiagen) and sequenced in both directions with the PCR primers and BigDye Terminator v3.1 Cycle Sequencing Kit (Applied Biosystems). External sequencing service was provided by Entelechon or LGC Genomics. Raw data and alignments are deposited at TreeBase under the accession code TB2:S15415 (http://purl.org/phylo/treebase/phylows/study/TB2:S15415), as well as at GenBank of the National Center for Biotechnology Information (NCBI); see S6 Table for accession numbers. All necessary permits were obtained for the described study, which complied with all relevant regulations. Specifically, the Gobierno de Canarias (#103.119), Cabildo de La Palma (#2011001784), Excmo. Cabildo Insular de La Gomera (#1578), and Cabildo de Tenerife (#27540) granted permits to collect plant specimens. DNA bank of the Gran Canarian flora at the Jardín Botánico Canario “Viera y Clavijo”, Las Palmas de Gran Canaria provided additional DNA samples from their collection.

### Lineage divergence estimates

We used BEAST V1.7.4/1.8.0 [18] to estimate divergence times between our MLF lineages and their putative closest relatives. Model parameters were GTR+Γ+I with 4 Γ categories and base frequencies were estimated. A relaxed clock model under an uncorrelated lognormal rate prior was chosen. Analyses used random starting trees and a speciation model following a birth—death process as tree prior. The number of generations was chosen such that the effective sample size (ESS) of all parameters was at least 200 (e.g. in an initial BEAST analysis the number of generations were set to 10 Mio, if the ESS was lower than 200, additional analysis with more generations were performed). Sampling of trees was done such that at least 10^3^ trees were available. Resulting posterior distributions for parameter estimates were checked in Tracer 1.4.1 [18] and maximum credibility trees, representing the maximum a posteriori topology, were calculated after removing burn-in (10% of the trees) with TreeAnnotator (version 1.4.7). Trees are deposited in Treebase.

### Calibration

Two approaches were used to calibrate the molecular clocks, depending on the data available. A) When reliable fossils were available, a lognormal prior on age estimates was used with an 2.5% quantile equal to the minimum age of the fossil; the 97.5% quantile was set to correspond to the maximum age assigned to a more basal, inclusive clade containing the group of interest (see S4 Table for calibration parameters for all species.). For example, the minimum age of Lauraceae was estimated using *Potomacanthus lobatus* fossil flowers from the middle Albian [19]. This fossil is contemporaneous with lauraceous leaf fossils from America and Europe [20], [21]. *Potomacanthus* shows the typical valvate anthers of Lauraceae and some related families. Because it does not only share the unique character set of Lauraceae, but also of related families [19] we placed this fossil at the stem node of Lauraceae. Accordingly, the offset was fixed to 106.4 such that the 2.5% quantile corresponded to the middle Albian (106.8 Ma stem node of Lauraceae). The upper bound of this node, represented by the 97.5% quantile, was set to 132.4 Ma, corresponding to the highest value of the published 95% Highest Posterior Densities (HPD) of Laurales (133–107 Ma) by Bell et al. (2010) [22].

B) In the absence of reliable fossils, published age estimates (e.g., family ages by Wikström et al., 2001 [23] and Bell et al., 2010 [22]) were applied to the corresponding nodes. For most taxa we used mean values of the older study by Wikström et al. (2001) [23] and combined them with those of Bell et al. (2010) [22] to consider the range of published ages, which in most cases did not strongly differ from each other. If available, we used age estimates derived from studies with denser taxon sampling (e.g. Bell & Donoghue, 2005) [24] for *Viburnum*/*Sambucus*). We modelled the entire range of the given mean age estimates as a normal distribution prior. Standard deviation was chosen such that the 2.5% quantiles corresponded to the lowest value of the 95% HPD by Bell et al. (2010) [22] for family ages or by Bell & Donoghue (2005) [24] in the case of *Viburnum*/*Sambucus*. As example, for *Ixanthus* the mean ages of the stem node of Gentianaceae were estimated to be 46, 52 and 52 Ma by Wikström et al. (2001) [23] and 50 and 53 Ma by Bell et al. (2010) [22] based on different methods. Means of both analyses were calculated (50.00 and 51.5 Ma). A mean value (50.75) of both was used as mean for the normal distribution. The standard deviation was set to 7.00 such that the 2.5% quantile (37.03) covered the lowest value of the 95% HPD (37 Ma) [22]. For other taxa (*Picconia*, *Rhamnus*) the 95% HPD were not available and we applied the same values of mean and standard deviation as given in previous publications (S4 Table). For Crassulaceae, the nrITS data set covered most of the island species of *Aeonium*, *Aichryson* and *Monanthes*. The inclusion of suitable taxa for calibration at crown node of Crassulaceae was not possible due to unavailability of material. Therefore, a secondary calibration was applied to the nrITS data using the dating outcome of a *mat*K data set. For some taxa, two calibration points were used.

### Ancestral states reconstruction

For the reconstruction of the ecological preference (laurel forest vs. non-laurel forest) and preadaptation of laurophyllous traits, we traced these features on phylogenetic trees using a likelihood criterion in Bayestraits V2.0. The number of outgroups and the number of BEAST trees differed across our data sets. To standardise the approach we regularly selected 1000 trees and focused only on clades including MLF taxa. Therefore, we trimmed the trees using the “droptip” function from the ‘ape’ package [25] in RStudio (2013) [26], so they only contained all Macaronesian laurel forest accessions and 10 operational taxonomic units (species) basal to this clade. As suggested by Pagel & Meade (2013) [27] for the reconstruction of ancestral states, we used Markov chain Monte Carlo (MCMC) methods to derive posterior distributions. MCMC allows the trait to change from its current state at any given moment to any other state over infinitesimally small intervals of time, consequently taking topological uncertainty into account. A multistate model was chosen to reconstruct how multiple traits evolve on phylogenetic trees. This option allowed us to incorporate several discrete traits into one analysis. We defined the nodes of the MLF taxa and their sister group with the AddMRCA command reconstructing the mrca to be present in all trees, simulating a possibly extinct ancestor. This allows the MRCA reconstruction to find the node in each tree in the sample that minimally contains all of the species or tips whose common ancestral state is of interest, even if this MRCA might include other species. Besides increasing the iterations to 10010000 and sampling frequency to 10000, default settings were used.

The following states were coded: A) habitat: 0 laurel forest vs. 1 non-laurel forest, B) leaves: 0 evergreen vs. 1 deciduous, C) habit: 0 woody vs. 1 herbaceous, D) life form: 0 annual vs. 1 perennial, E) leaf margin: 0 entire vs. 1 non-entire. Attribution to a certain state was based on literature data (cf. S5 Table, data matrix available on request from authors) and herbarium material. For example, the state of a laurel forest habitat was assigned to a species if it strictly occurred in this forest type. In some cases, where a clear assignment was not possible, the state was defined as a missing character (“-“). Traits with a P value over 0.6 are treated as positive, lower than 0.4 as negative and the rest as ambiguous indication for the corresponding character.

### Biogeographical analyses

We applied ancestral area reconstructions based on the dispersal-extinction-cladogenesis (DEC) model [28]. As input tree for Lagrange (V20130526) [28] we used the maximum clade credibility tree of the 1000 trimmed trees as used for the BayesTraits analyses. A Python script was created using the online Lagrange configurator (http://www.reelab.net/lagrange/con-figurator/index). Worldwide species distributions were categorised into six areas: Macaronesia, including the Canaries, Madeira and the Azores (= M), Europe and the Mediterranean region (= E), sub-Saharan Africa (= F), Asia (= S), the Americas (= A) and Australia (= U). All combinations of areas were allowed in the adjacency matrix, and baseline rates of dispersal and local extinction were estimated. To reduce the number of possibilities we limited the total number of combined areas to two. Ancestral area estimates are given for the stem node of MLF lineages and their sister clade/group. Only relative probabilities above 10% were considered to define possible ancestral areas of a MLF lineage. For each defined ancestral area(s) we added all relative probabilities across all analysed taxa and calculated their relative proportion relative to all areas.

## Results

### Phylogenies

Our phylogenetic results are in accordance with published phylogenies (S5 Table). Most of the analysed MLF species, or clades in the case of *Bystropogon* and *Isoplexis*, were found to be monophyletic. Exceptions are: *Aichryson pachycaulon*, which appears to be intermixed with other species, *Euphorbia stygiana* is weakly supported as paraphyletic to *E*. *mellifera*, *Isoplexis canariensis* appears to be polyphyletic, *Laurus azorica*, *L*. *novocanariensis* and *L*. *nobilis* from Morocco appear intermingled in the phylogeny (cf. Rodríguez‐Sánchez et al., 2009 [29]). Support values for MLF and other clades vary throughout the single phylogenies (see Treebase). Overall, different biogeographic patterns were revealed: In *Prunus* and *Viburnum*, Azorean taxa occupy a basal position within Macaronesian/Mediterranean clades. As for *Rhamnus*, *Sambucus*, *Laurus*, *Isoplexis* and *Euphorbia*, Mediterranean/European species are paraphyletic with respect to Macaronesian lineages. While *Picconia*, *Ixanthus* and *Arbutus canariensis* have their sister groups in the Mediterranean/Europe, the sisters of *Persea indica*, *Apollonias* and possibly *Ocotea foetens*, are of tropical distribution. Close relationships of MLF taxa to other Macaronesian taxa can be found among *Pleiomeris*, *Heberdenia*, *Bystropogon*, *Aeonium* and *Aichryson*.

### Molecular dating

**Fig 2:**
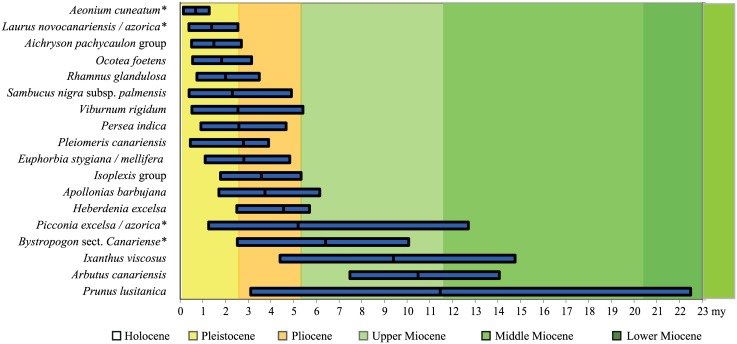
Stem age estimates of the studied laurel forest species (Ma: million years ago). Bars show the 95% HPD, mean stem ages are marked by a vertical black line. Asterisks (*) indicate taxa whose mrca habitat is not optimised as laurel forest.

The mean stem ages (Fig 2 and Table 1) of all taxa range from 11.5 to 0.7 Ma. In 14 of the 18 studied lineages they fall into the Plio-Pleistocene, in *Prunus lusitanica*, *Arbutus canariensis*, *Ixanthus viscosus* and *Bystropogon* sect. *Canariense* into the late Miocene (11.6–5.3 Ma). The 95% HPD vary from 22.5 to 0.1 Ma. *Prunus lusitanica* including all its subspecies is the oldest taxon with the 95% HPD extending to the early Miocene (23–16 Ma). The subspecies are much younger, the split between *Prunus lusitanica* subsp. *azorica* and subsp. *hixa*/subsp. *lusitanica* is dated to 8.0–0.6 Ma (95% HPD) with a mean stem age of 3.53 Ma. The upper 95% HPD values of *Arbutus canariensis*, *Picconia excelsa*/*azorica* and *Ixanthus viscosus* reach into the middle Miocene (16.0–11.6 Ma). In 13 taxa the 95% HPD overlap in the Plio-Pleistocene transition at 2.6 Ma.

**Table 1:** Stem age estimates of MLF taxa using BEAST as means and 95% highest posterior densities (HPD) in Ma.

Taxon	Lower 95% HPD value	Upper 95% HPD value	Mean stem age
*Prunus lusitanica*	3.11	22.49	11.46
*Arbutus canariensis*	7.48	14.06	10.48
*Ixanthus viscosus*	4.40	14.76	9.40
*Bystropogon* sect. *Canariense*	2.52	10.07	6.41
*Picconia excelsa*	1.26	12.70	5.20
*Heberdenia excelsa*	2.50	5.70	4.56
*Apollonias barbujana*	1.71	6.15	3.74
*Isoplexis* group	1.78	5.33	3.58
*Prunus lusitanica* subsp. *azorica* and *hixa* / *lustanica*	0.59	8.01	3.53
*Euphorbia mellifera / stygiana*	1.10	4.83	2.81
*Pleiomeris canariensis*	0.45	3.90	2.79
*Persea indica*	0.91	4.67	2.59
*Viburnum rigidum*	0.52	5.41	2.55
*Sambucus nigra* subsp. *palmensis*	0.39	4.91	2.30
*Rhamnus glandulosa*	0.74	3.49	2.00
*Ocotea foetens*	0.55	3.15	1.83
*Aichryson pachycaulon* agg.	0.51	2.70	1.49
*Laurus novocanariensis / azorica*	0.39	2.55	1.39
*Aeonium cuneatum*	0.14	1.28	0.68

### Habitat and trait optimisation

We tested whether the ancestral habitat of our studied MLF taxa and their sister was a laurel forest. The resulting posterior probabilities (P) for the non-laurel forest habitats varied from 0.37 to 0.95. A laurel forest origin was rejected for *Aeonium cuneatum*, *Laurus novocanariensis/azorica*, *Picconia excelsa/azorica*, *Arbutus canariensis* and *Bystropogon* sect. *Canariense*. For 12 taxa the results were ambiguous (Table 2, Fig 2).

**Table 2:** Results of BayesTraits analyses concerning laurel forest ecology (columns 2–3) and morphological traits (columns 4–11).

MRCA of	P (LF)	P (not LF)	P (not Ev)	P (Ev)	P (H)	P (W)	P (Pn)	P (A)	P (not En)	P (En)
*Aeonium cuneatum*	*0*,*06*	**0,95**	**1,00**	*0*,*00*	0,47	0,53	**1,00**	*0*,*00*	*0*,*00*	**1,00**
*Aichryson pachycaulon* group	0,50	0,50	0,50	0,50	**0,89**	*0*,*11*	*0*,*16*	**0,85**	*0*,*00*	**1,00**
*Apollonias barbujana*	0,53	0,47	*0*,*01*	**0,99**	*0*,*00*	**1,00**	**1,00**	*0*,*00*	*0*,*00*	**1,00**
*Arbutus canariensis*	*0*,*37*	**0,63**	*0*,*00*	**1,00**	0,47	0,54	**1,00**	*0*,*00*	0,50	0,50
*Bystropogon* sect. *Canariense*	*0*,*39*	**0,61**	**0,74**	*0*,*26*	**0,83**	*0*,*17*	*0*,*05*	**0,95**	0,49	0,51
*Euphorbia stygiana/mellifera*	0,53	0,47	**0,73**	*0*,*28*	0,52	0,48	**1,00**	*0*,*00*	*0*,*32*	**0,68**
*Heberdenia exselsa*	0,60	0,40	*0*,*13*	**0,87**	*0*,*00*	**1,00**	**0,98**	*0*,*02*	*0*,*07*	**0,93**
*Isoplexis* group	0,50	0,50	*0*,*36*	**0,64**	0,50	0,50	**1,00**	*0*,*00*	0,49	0,51
*Ixanthus viscosus*	0,47	0,53	0,47	0,53	0,50	0,50	0,50	0,50	*0*,*00*	**1,00**
*Laurus novocanariensis/azorica*	*0*,*05*	**0,95**	*0*,*00*	**1,00**	*0*,*00*	**1,00**	**1,00**	*0*,*00*	*0*,*00*	**1,00**
*Persea indica*	0,44	0,56	*0*,*00*	**1,00**	*0*,*00*	**1,00**	**1,00**	*0*,*00*	*0*,*00*	**1,00**
*Picconia excelsa/azorica*	*0*,*23*	**0,77**	*0*,*06*	**0,94**	*0*,*00*	**1,00**	**1,00**	*0*,*00*	0,42	0,59
*Pleiomeris canariensis*	0,53	0,47	*0*,*13*	**0,87**	*0*,*00*	**1,00**	**0,98**	*0*,*02*	*0*,*07*	**0,93**
*Prunus lusitanica*	0,49	0,51	0,57	0,44	*0*,*00*	**1,00**	**1,00**	*0*,*00*	**0,96**	*0*,*04*
*Rhamnus glandulosa*	0,44	0,56	*0*,*09*	**0,91**	*0*,*00*	**1,00**	**1,00**	*0*,*00*	**0,96**	*0*,*04*
*Sambucus nigra* subsp. *palmensis*	0,51	0,50	**1,00**	*0*,*00*	*0*,*31*	**0,70**	**1,00**	*0*,*00*	**1,00**	*0*,*00*
*Viburnum rigidum*	0,55	0,45	*0*,*13*	**0,87**	*0*,*00*	**1,00**	**1,00**	*0*,*00*	*0*,*06*	**0,94**

MRCA: most recent common ancestor; P: Posterior probability; LF: laurel forest; Ev: evergreen; H: herbaceous; W: woody habit; Pn: perennial; A: annual; En: entire leave margin. P value > 0.6 in bold letters., < 0.4 in italics.

**Fig 3:**
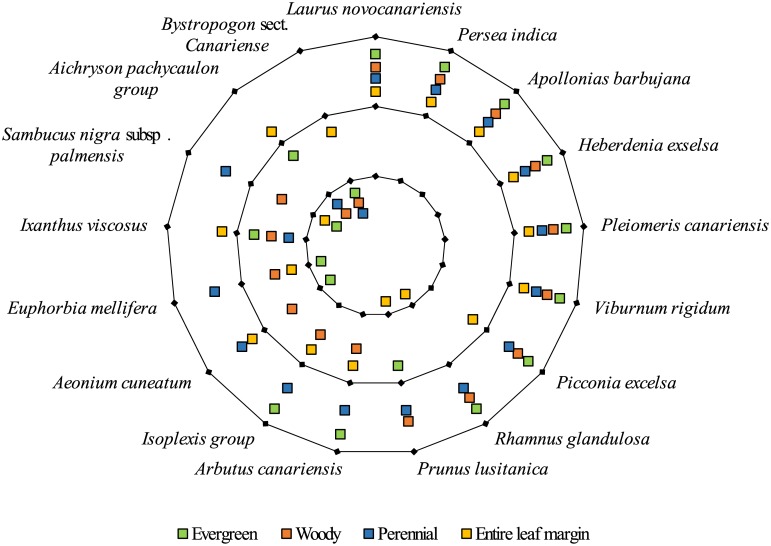
Optimisation of morphological traits to the mrca of the MLF species/sister in a radar plot. In the outer circle laurophyllous traits are supported by BayesTraits analyses, in the inner circle they are rejected, in the middle circle the optimisation is ambiguous. The cutting value for the marginal probability: > 0.6, accepted; 0.6–0.5, ambiguous; < 0.4, rejected.

Morphological trait reconstructions were conducted to test a possible pre-adaptation concerning laurophylly of MLF ancestors. A woody and perennial habit, evergreen leaves and entire leaf margins were considered. The results revealed the full combination of these characters for the mrca of seven of the 18 taxa (Table 2 and Fig 3). In six cases the mrca were optimised having three or two traits. Nine taxa were optimised with two, one or none of these traits. *Bystropogon* sect. *Canariense*, *Aichryson pachycaulon* agg. and *Sambucus nigra* subsp. *palmensis* showed the highest likelihood of not having laurophyllous traits.

### Biogeographical analyses

**Fig 4:**
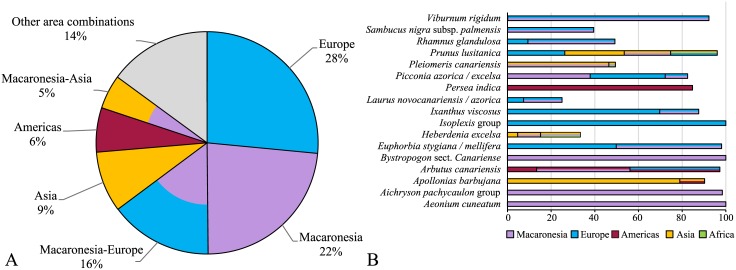
Source areas of Macaronesian laurel forest taxa as indicated by Lagrange. A) Proportion of area or area combinations as ancestral area for MLF based on relative probabilities over all taxa. B) Area reconstruction of each taxon with a likelihood > 10%. X-axis represents relative probabilities. Colours: (lilac) Macaronesia (Azores, Madeira, Canary Islands), (light blue) Europe incl. Mediterranean, (yellow) Asia, (dark red) North and South America, (green) Africa.

Relative probabilities for the ancestral area/combinations of areas of the corresponding node are listed in S7 Table and visualised in Fig 4. A common source area for all MLF taxa could not be detected. The European/Mediterranean (28%), the Macaronesian (22%) and the combined European-Macaronesian (16%) regions were revealed as ancestral areas with the highest probability. *Aeonium cuneatum*, *Aichryson pachycaulon* agg., *Bystropogon* sect. *Canariense* likely originated in Macaronesia, *Isoplexis* in Europe and *Persea indica* in the Neotropics. The distributions of the mrca of *Viburnum rigidum*, *Sambucus nigra* subsp. *palmensis*, *Rhamnus glandulosa*, *Picconia excelsa/azorica*, *Ixanthus viscosus* and *Euphorbia stygiana/mellifera* and their respective sister were optimised to the combined area of Macaronesia and Europe. Other apparent sources are Macaronesia-Asia (*Pleiomeris*) and tropical Asia (*Apollonias*). The origin of the remaining four species was ambiguous and included at least three different geographical areas.

## Discussion

The stem node ages of our analysed MLF lineages range from the early/middle Miocene to the Pleistocene with a cluster in the Plio-Pleistocene. The ancestral habitat reconstruction (laurel forest vs. non-laurel forest) did not reveal significant outcomes for most of the species. The biogeographic analyses revealed different source areas of MLF taxa.

### Relict hypothesis

The test if the MLF are relictual is not trivial. Our age estimates date the stem nodes of our studied MLF taxa to the Neogene and Quaternary (2.6–0 Ma) and rule out a Paleogene origin. Inferring from 95% HPD intervals, the large majority of our taxa originated in the Plio-Pleistocene. This period spans the time when Mediterranean laurel forests were mostly extinct. The disappearance of continental, laurophyllous elements was not a sudden event, but rather a slow process of decline. According to Mai (1989) [15], these forests were at the latest extinct at the Plio-Pleistocene transition. Single laurophyllous elements may have sporadically survived in small, climatically suitable patches beyond the Plio-Pleistocene boundary. Some populations of *Ocotea* in the Mediterranean may have survived until the early Pleistocene (2.6–0.8 Ma; S3 Table) [30]. This shows that the Plio-Pleistocene boundary is not a fixed point to test a relict origin of MLF. Overall, laurophyllous forests were relatively rare across Europe and the Mediterranean already in the Pliocene. This sparsity does not exclude, but reduces the chance that they were the source vegetation of MLF. Different types of Atlantic Ocean relicts have been described by Cronk (1992) [31] distinguishing between ancient (> 10 Ma), subancient (late Miocene/Pliocene) and recent relicts from the Pleistocene. Our data confirm varying ages of MLF taxa, however, clustering to Plio-/Pleistocene and late Miocene.

### Plio-/Pleistocene taxa

Due to young ages and ecological preferences (non-laurel forests) of closely related taxa, a relict status is rather unlikely for several of our MLF taxa, such as *Aeonium*, *Aichryson*, *Rhamnus*, *Viburnum*, *Sambucus*, *Persea* and *Euphorbia*. They originated in the Plio-Pleistocene. Of similar age are MLF species of *Echium* (3.9 Ma) [32], *Erica arborea* (2.9 Ma) [33] and *Trechus* beetles (Carabidae) [34]. The Pleistocene age of the Macaronesian *Laurus novocanariensis*/*azorica* and *Ocotea foetens* does not favour the ‘Tertiary relict’ hypothesis either. The closest relative of *Laurus novocanariensis*/*azorica*, the Mediterranean *Laurus nobilis*, today occurs in wetter parts of deciduous forests and also in disturbed places. The insular *Laurus* taxa could nonetheless be interpreted as relicts, if *L*. *nobilis* was a representative of Mediterranean laurel forests in the Pliocene. The same applies to *Ocotea foetens* if this taxon were closely related to the described Mediterranean *Ocotea* fossils from the late Plio- and early Pleistocene [30] and not to other tropical lineages of this speciose genus. Instead of being ‘Tertiary relicts’ they originated in relatively recent times as a result of repeated glaciations (see also below).

### Possible relicts

Some of our MLF taxa appear to be relatively old and could be considered as ‘Tertiary relicts’. The oldest groups are *Prunus lusitanica*, *Arbutus canariensis*, *Ixanthus*, *Bystropogon* sect. *Canariense* and *Picconia* with mean ages falling into the late Miocene and early Pliocene, and 95% HPD extending to the middle Miocene. Despite their relative old age, *Arbutus canariensis* and *Bystropogon* sect. *Canariense* do not fulfil the criteria of the ‘Tertiary relict’ hypothesis because they likely originated in the Americas and in Macaronesia itself, respectively. Inferring from relatively basal phylogenetic positions and partly from molecular dating, other putative old elements of MLF are *Ilex canariensis* (Aquifoliaceae) [35], *Morella faya* (Myricaceae) [36], *Visnea mocanera* (Pentaphylacaceae; Schüßler *et al*. in prep.), *Trichomanes speciosum* (Hymenophyllaceae, Oligocene), *Hymenophyllum tunbrigense* (Hymenophyllaceae, Paleogene) [37], *Pteris incompleta* (Pteridaceae, early Miocene) [38], *Hedenasiastrum percurrens* (Brachytheciaceae, Eocene), *Rhynchostegiella* species (Brachytheciaceae, late Miocene) [39] and the laurel pigeon, *Columba junoniae* (Columbidae) [40]. In the case of *Visnea*, preliminary molecular studies show that the MLF species, *V*. *mocanera*, is sister to other Asian-American genera (Schüßler *et al*. in prep.). The actual sister group could nonetheless be the fossil *Visnea germanica* which was widespread in the Mediterranean/Europe in the Mio-Pliocene [13]. This may affect the phylogenetic pattern found here, which is based on only recent taxa. A Macaronesian-Mediterranean relationship between *Visnea mocanera* and *V*. *germanica* would support the relict hypothesis. This case also exemplifies the problem of extinction in our analyses, which removes the signal from past speciation events, potentially obscuring the pattern. At the same time extinctions may argue against the relict hypothesis if undetected, non-laurel forest sister groups of MLF species died out. In parametric methods such as DEC, biogeographic events like extinction are modelled as stochastic processes evolving along branches, so these events are more likely to occur on longer branches than on shorter ones [41]. Our BayesTraits analyses, testing if laurel forests were the ancestral habitat of our taxa are, however, inconclusive. The age estimates neither allow a definite statement on the relict status in most of the cases.

### Macaronesian-Mediterranean patterns

A few present-day species like *Woodwardia radicans*, *Erica scoparia* and *Prunus lusitanica* occur in the Mediterranean and in MLF. According to the relict hypothesis, the continental populations could be survivors of the Neogene laurophyllous vegetation or they were extinct and re-colonised the mainland in more recent times. The diversification of *Prunus lusitanica* into subsp. *azorica* (Azores) and subsp. *hixa* (Canaries, Madeira)/subsp. *lusitanica* (Iberia and Morocco) is dated around the Pliocene (late Miocene to Pleistocene). Importantly, subsp. *azorica* occupies a more basal position than the mainland populations. This is a pattern which is supported by the higher genetic variation in Azorean populations than in continental ones as found for *Prunus lusitanica* [42] and *Erica scoparia* [43]. Islands have likely served as refugia during the Pleistocene, where multiple colonisations and/or allopatric differentiation has contributed to high levels of genetic diversity. Dispersal to the continent, extinction (e.g., fossil *Prunus lusitanica* in Italy [44]), and postglacial range expansion explain the observed biogeographic patterns [43]. For *Laurus*, the variation of cpDNA haplotypes [29], AFLP data [45] and the relatively high morphological variation with few distinctive characters [11] likely also indicate complex Pleistocene dynamics in the Mediterranean and Macaronesia. This is compatible with our dating.

### Macaronesian fossil record

The Macaronesian fossils attributed to the MLF date to the Pliocene and Pleistocene [46], which coincides with our proposed dates of origin. Heer´s (1857) [47] fossils from the Pleistocene in Madeira are likely closely related to modern species, as in the case of *Ocotea*. Anderson et al. (2009) [46] reported Pliocene fossils from Gran Canaria attributed to *Ilex* and *Arbutus*, both old elements, *Hedera* and putative Lauraceae. Whether these laurophyllous fossils are progenitors of the modern species (for Lauraceae, they are likely *Persea* or *Apollonias* according to our data) or belong to other lineages cannot be evaluated based on the available “fossil” morphological characters. According to the fossil evidence, laurophyllous elements existed on the islands at least since the Pliocene. Whether these were already forming forests, in the sense of a vegetation type, cannot be decided.

Given all our results, none of the criteria for the relict hypothesis is entirely met. MLF seem to have a rather heterogeneous origin in terms of their spatial and temporal patterns. In conclusion, we reason that MLF contain some old, possibly relict taxa, though many taxa are likely not ‘Tertiary relicts’. The youngest species of today’s MLF date to the Pleistocene. Consequently, the MLF in its present species composition is of Pleistocene origin.

### Impact on taxonomy of fossils

Our age estimates differ from many fossils attributed to European laurel forests. For example, *Laurus* has been reported from the Eocene to Pleistocene (S3 Table) [12]. *Laurus abchasica* from the early Miocene to Pliocene has been purported to be the direct ancestor of all extant *Laurus* species [11]. This is partly supported by our data indicating a stem node age for the genus *Laurus* of 8.2–2.9 Ma. Our date suggests that the determination of specimens as *Laurus*, which are older than 8.2 Ma, should be treated with caution (cf. Worobiec, 2007 [12]). Moreover, we doubt whether a single species, *Laurus abchasica*, existed over such a long period. As the taxonomy of recent Lauraceae is often debated [48], it is plausible to assume similar problems for the fossils. Therefore, an attribution of pre-upper Miocene fossils to other extinct lineages should be considered. Several of such lauroid fossils have been grouped to the artificial genus ‘*Laurophyllum*’.

### Species turnover/new species assembly

Scenarios explaining the tight clustering of the age estimates in the Plio- and Pleistocene largely depend on palaeoclimatic data. Fernández‐Palacios et al. (2011) [3] proposed a suitable climate for laurel forests in the late Miocene to middle Pliocene (11.6–3.0 Ma). Accordingly, MLF may have already existed in the islands since the Miocene (or earlier) and undergone a progressive turnover in their species composition. The relatively old age of some of our studied and the above cited species support the ‘Tertiary relict’ hypothesis. The simultaneous occurrence of taxa from the Plio-/Pleistocene indicates dynamics in these forests. In this case, an intense replacement of the older vegetation by the present-day one would have started in the Pliocene. Laurophyllous fossils older than the Pliocene have not (yet) been found in Macaronesia.

The palaeoclimatic data are, however, incomplete. Based on dust proxy, isotopes and organic carbon analyses the trade winds on the Canary Islands were enhanced at terminal time periods of the late Pleistocene glacials [8], [9]. Therefore, sufficient water supply could have been established with the trade wind regime in the Pleistocene. At the same time, diminished trade winds may have precluded the formation of the MLF prior to the Pleistocene, perhaps before the Pliocene. It is not clear if and how trade winds determined the occurrence of MLF before the Pleistocene. In the case that trade winds were a new phenomenon on the islands, MLF may also represent a novel vegetation type. They were likely influenced by interglacials which may have pushed the forest species through genetic bottlenecks. Species communities and vegetation types can be very dynamic through space and time (e.g. Overpeck et al., 1992 [49]). Alternatively, a combination of species turn-over and repeated expansion-extinction processes might be possible. Pliocene or even Miocene MLF could have harboured different species through time and could have gained new taxa in the Pleistocene due to suitable conditions linked with the enhancement of trade winds.

### Geographical sources and pre-adaptation

Our data suggest that the constituent MLF species were recruited from different source areas, including the Mediterranean, Macaronesia and the Palaeo- and Neotropics. We support the affinities to the Mediterranean region, as postulated by Carine et al. (2010) [50]. Several taxa, however, originated in Macaronesia itself. This applies to the island radiations of Crassulaceae, *Bystropogon* and *Echium* and other taxa. In contrast to our results, American affinities of *Apollonias barbujana* were indicated based on a larger taxon sample within this group and the use of other DNA markers [51]. *Persea indica* likely originated from American tropical lineages, respectively. This global recruitment could be a general pattern, demonstrated for the Cape flora [52], Hawaii [53] and the Southern Hemisphere floras in general [54]. Besides relatedness and geographical proximity to Mediterranean type laurel forests, other factors may have influenced the sourcing of MLF. In several cases, the mrca of the MLF species/sister already had the full or nearly complete syndrome of these typical traits (Fig 3). The morphological differences to sclerophylly are relatively few and mainly found in the amounts of sclerenchyma, leaf shape, and margins. Therefore, the conservative state of laurophylly is not surprising. The role of pre-adaptations is consistent with findings from many different groups, from different areas [55].

## Conclusions

Our age estimates, habitat reconstructions, the heterogeneous biogeographic histories and the fossil record indicate that the majority of our analysed MLF taxa should not be considered as relicts of a formerly widespread Mediterranean ‘Tertiary vegetation’. MLF, in their present angiosperm composition, are relatively young plant communities recruited from European/Mediterranean and tropical regions. Important factors for the colonisation of the MLF were recurrent dispersal over medium and long distances, morphological preadaptation and ecological adaptation. This illustrates that at least parts of the Mediterranean biodiversity hotspot are likely much younger than previously thought [56, 57], and either underwent a large species turn-over or were newly composed as suitable climates were available. Our results should, however, not question the conservation status of MLF due to their unique and endemic species composition. The relict condition might be just a matter of scale: although these forests are presumably not ‘Tertiary relicts’, they represent remnants of the Pliocene/Pleistocene forests in Macaronesia that likely underwent expansions and reductions in their distribution areas during the Pleistocene temperature oscillations. Today, plants introduced and native to Europe like *Prunus laurocerasus*, *Ilex aquifolium*, *Rhododendron ponticum*, *Hedera helix*, *Trachycarpus fortunei* and *Arbutus unedo* tend to expand their range [58]. This indicates that similar processes which acted during the formation of MLF, still to seem be active. These are possibly playing a role in the creation of new laurophyllous forests outside of classical areas, recruiting elements from diverse source areas.

## Supporting Information

S1 Table Chronology of the history of the relict hypothesis for MLF vegetation. (PDF)

S2 Table Genera of Macaronesian laurel forest plants with their distribution on the archipelagos. (PDF)

S3 Table Fossil record of selected genera representing laurophyllous vegetation of Europe and Macaronesia from the Neogene and Paleogene. (PDF)

S4 Table Calibration points and variable parameters used for the BEAST analyses. In the second column applied ages and their source are stated. m: mean; s: standard deviation; mn: minimal age; mx: maximum age; loHPD: lowest value of 95% HPD.
(PDF)

S5 Table Published phylogenies of the studied taxa. (PDF)

S6 Table Taxa used for molecular analyses and Genbank accession numbers. New sequences for this study are marked in italics.
(PDF)

S7 Table Results of the Ancestral area reconstruction using Lagrange. Coded regions: E: Europe/Tethys, M: Macaronesia, S: Asia, A: North & South America, F: Africa; Rel. Prob.: relative probability. Estimates above 10% are marked bold.
(PDF)
