# Supplementary material for: Biogeography of Mediterranean Hotspot Biodiversity: Re-Evaluating the 'Tertiary Relict' Hypothesis of Macaronesian Laurel Forests
Source: PLoS One. 2015 Jul 14;10(7):e0132091. doi: 10.1371/journal.pone.0132091 (PMC4501571; doi:10.1371/journal.pone.0132091)
Supplement: S1 Table — (PDF) [file pone.0132091.s001.pdf]

S1 Table. Chronology of the history of the relict hypothesis for MLF vegetation.

**Citation**	**Thesis**	**Postulated age of MLF**	**Evidence**
Heer 1857 [1]	The fossils of St. Jorge (Madeira) are related to recent laurel forest elements of Madeira indicating a certain degree of species turnover. They represent a Pleistocene vegetation.	Pleistocene	Fossils
Heer 1860 [2]	There is a close relationships between *Laurus novocanariensis* (recent) and *L. princeps* (Upper Miocene).	Pleistocene	Fossils
Hooker 1867 [3] see Williamson 1984 [4]	Parts of present-day Macaronesian flora are relicts of European Tertiary flora.	Tertiary (Palaeogene & Neogene)	European fossils (Heer, 1857)
Engler 1879 [5]	Parts of present-day Macaronesian flora are relicts of European Tertiary flora.	Tertiary, implicit Upper Miocene (Heer 1860), explicitly at least since Pleistocene	Fossils (e.g., Laurus princeps see Heer 1860), distribution patterns, ecology
Schenck and Schimper 1907 [6]	The Macaronesian laurel forest is a relict of the European Miocene-Pliocene forest.	Miocene, Pliocene	Fossils
Ehrendorfer 1970 [7]	Laurel forest elements migrated from Asia into Europe/Mediterreanean possibly in early Tertiary. Macaronesian laurel forests are relicts of Middle Tertiary forests.	Middle Tertiary	Chromosome numbers; distribution areas
Mai 1971 [8]	Part of Macaronesian laurel forest genera were present in Central Europe during the Tertiary.	Eocene till Miocene (Central Europe)/Pliocene (South-Europe)	Fossil fruits/seeds
Ferguson 1974 [9]	*Laurus abchasica* is basal to *L. nobilis* and *L. novocanariensis.*	Pliocene	Fossils, morphology
Axelrod 1975 [10]	Macaronesian laurel forest originated from northern African Middle Miocene forests.	Middle Miocene	Fossil floras, distribution areas, plate tectonics
Bramwell 1976 [11]	Macaronesian laurel forest is relict of the European Miocene-Pliocene forest.	Miocene, Pliocene	Fossils

Citation	Thesis	Postulated age of MLF	Evidence
Takhtajan 1978 [12]	Macaronesian laurel forests are similar to European evergreen forests in the Miocene/Pliocene.	Miocene, Pliocene	Reconstruction of former floras; review of carpological and leaf fossils
Sunding 1979 [13]	Several Macaronesian endemics are late Tertiary relicts.	Miocene, Pliocene	Fossils
Takhtajan *et al.* 1986 [14]	Macaronesian laurel forest is closely related to European and Caucasian late Miocene/Pliocene evergreen forests.	Late Miocene/Pliocene	Distribution areas, systematic positions
Mai 1989 [15]	Laurophyll vegetation in Europe since Early Tertiary. Differentiation in Eocene. Decline since Oligocene. Extinction before Pleistocene. Parts of present-day Macaronesian flora are relicts of European Tertiary laurophyll flora.	Tertiary	Fossil floras in Europe
Bramwell *et al.* 1990 [16]	Parts of present-day Macaronesian flora are relicts of European Tertiary flora.	Tertiary, min. Miocene	Lauroid leaf fossils from the Miocene and Pliocene in the Mediterranean region (no citation!)
Cronk 1992 [17]	Parts of present-day Macaronesian and St. Helenas flora are relicts of African (and other) Tertiary flora; three main relict types: (1) Miocene age/ancient relicts (e.g. *Picconia*, *Apollonias*); (2) late Miocene or Pliocene/subancient relicts, (3) late Pliocene/recent relicts.	Miocene	Fossils outside from Macaronesia similar to "relict" species
Mai 1995 [18]	Laurel forests were widespread in the Eocene, Oligocene and Middle & Upper Miocene and declined since Oligocene.	Tertiary	Fossil floras
Morales *et al.* 1996 [19]	The Macaronesian laurel forest is a relict forest of the Tertiary Mediterranean flora, which occupied southern Europe and northern Africa about 20 Ma.	Tertiary, about 20 Ma	No evidence/ citation mentioned

Citation	Thesis	Postulated age of MLF	Evidence
Sjögren 2000 [20]	In general one must consider the Macaronesian flora as very old and even nonendemic species are in many cases of relictic nature. (*Ocotea*, *Viburnum*, *Clethra*, *Laurus*, *Ilex*, *Persea*, *Picconia* and the fern *Woodwardia*).	Tertiary	Tertiary relicts in Macaronesia; fossils (no explicit citation)
Whittaker and Fernández-Palacios 2007 [21]	Macaronesian laurel forest species might be palaeoendemics, which were widespread in the Tertiary Mediterranean flora.	Min. late Pliocene /min. 2 Ma, very likely much older	Fossils from Europe (Axelrod 1975, Sunding 1979)
Vargas 2007 [22]	Macaronesian laurisilva are surviving representatives of the Tethyan-Tertiary flora	Tertiary, Miocene/Pliocene	Bramwell 1976
Calleja *et al.* 2009 [23]	The disjunct distribution is a result of the fragmentation of the Tertiary European subtropical lauroid vegetation during the Plio-Quaternary climate change.	Tertiary relict	Leaf morphology (adaptation to summer drought); part of macLF; disjunct distribution
Anderson *et al.* 2009 [24]	Fossils - of which the determination is partly yet to be confirmed - found on Gran Canaria indicate presence of laurel forest in Pliocene (/Miocene).	Miocene-Pliocene	Various kinds of macrofossils
Postigo Mijarra *et al.* 2009 [25]	During the Piacenzian evergreen laurel forests (7 Lauraceae) disappeared almost completely from the Iberian Peninsula.	Oligocene-Piacenzian (3.6-2.6)	Pollen, spores and leaf and wood remains
Fernández-Palacios *et al.* 2011 [26]	Some species survived on Macaronesian islands but became extinct in Iberia, e.g. *Apollonias*, *Clethra*, *Ocotea*, *Persea*, *Picconia*, *Pleiomeris*, *Visnea*.	Before Pliocene	Fossils from Europe

1. Heer O (1857) Über die fossilen Pflanzen von St. Jorge in Madeira. Neue Denkschriften der Allgemeinen Schweizerischen Gesellschaft für die Gesammten Naturwissenschaften 15: 1-40.
2. Heer O (1860) Untersuchungen über das Klima und die Vegetationsverhältnisse des Tertiärlandes. Winterthur Wurster & Comp.

3. Hooker JD (1867) On insular floras. Gardeners' Chronicle: 6-7, 27, 50-51, 75-76.
4. Williamson M (1984) Sir Joseph Hooker's lecture on insular floras. Biological Journal of the Linnean Society 22: 55-77.
5. Engler A (1879) Versuch einer Entwicklungsgeschichte, insbesondere der Florengebiete seit der Tertiärperiode. I. Die extra-tropischen Gebiete der nördlichen Hemisphäre. Leipzig: Engelmann.
6. Schenck H, Schimper AFW (1907) Beiträge zur Kenntniss der Vegetation der Canarischen Inseln. Wissenschaftliche Ergebnisse der Deutschen Tiefsee-Expedition auf dem Dampfer "Valdivia" 1898-1899 2: 232-393.
7. Ehrendorfer F (1970) Mediterran-mitteleuropäische Florenbeziehungen im Lichte cytotaxonomischer Befunde. Feddes Repertorium 81: 3-32.
8. Mai DH (1971) Über fossile Lauraceae und Theaceae in Mitteleuropa. Feddes Repertorium 82: 313-342.
9. Ferguson DK (1974) On the taxonomy of recent and fossil species of *Laurus* (Lauraceae). Botanical journal of the Linnean Society 68: 51-72.
10. Axelrod DI (1975) Evolution and biogeography of Madrean-Tethyan sclerophyll vegetation. Annals of the Missouri Botanical Garden 62: 280-334.
11. Bramwell D (1976) The endemic flora of the Canary Islands. In: Kunkel G, editor. Biogeography and Ecology in the Canary Islands. The Hague: Junk. pp. 207-240.
12. Takhtajan A (1978) Floristic regions of the world. Leningrad: Nauka.
13. Sunding P (1979) Origins of the Macaronesian flora. In: Bramwell D, editor. Plants and islands. London: Wiley. pp. 13-40.
14. Takhtajan A, Crovello TJ, Cronquist A (1986) Floristic regions of the world. Berkeley, Los Angeles, London: University of California Press. 522 p.
15. Mai DH (1989) Development and regional differentiation of the European vegetation during the Tertiary. Plant Systematics and Evolution 162: 79-91.
16. Bramwell D, Croker EO, Sánchez-Pinto L, Bramwell ZI (1990) Flores silvestres de las Islas Canarias. Editorial Rueda: Madrid
17. Cronk Q (1992) Relict floras of Atlantic islands: patterns assessed. Biological Journal of the Linnean Society 46: 91-103.
18. Mai D (1995) Tertiäre Vegetationsgeschichte Mitteleuropas. Fischer, Jena.
19. Morales D, Jiménez MS, González-Rodríguez AM, Cˇermák J (1996) Laurel forests in Tenerife, Canary Islands. Trees 11: 34-40.
20. Sjögren E (2000) Aspects on the biogeography of Macaronesia from a botanical point of view. Arquipélago Life and Marine Sciences Supp. 2 (A): 1-9.
21. Whittaker RJ, Fernández-Palacios JM (2007) Island biogeography: ecology, evolution, and conservation. New York Oxford University Press.
22. Vargas P (2007) Are Macaronesian islands refugia of relict plant lineages?: a molecular survey. Phylogeography of southern European refugia: Springer. pp. 297-314.
23. Calleja J, Benito Garzón M, Sáinz Ollero H (2009) A Quaternary perspective on the conservation prospects of the Tertiary relict tree *Prunus lusitanica* L. Journal of Biogeography 36: 487-498.
24. Anderson CL, Channing A, Zamuner AB (2009) Life, death and fossilization on Gran Canaria–implications for Macaronesian biogeography and molecular dating. Journal of Biogeography 36: 2189-2201.
25. Postigo Mijarra JM, Barrón E, Gómez Manzaneque F, Morla C (2009) Floristic changes in the Iberian Peninsula and Balearic Islands (south-west Europe) during the Cenozoic. Journal of Biogeography 36: 2025-2043.
26. Fernández-Palacios JM, de Nascimento L, Otto R, Delgado JD, García-del-Rey E, et al. (2011) A reconstruction of Palaeo-Macaronesia, with particular reference to the long-term biogeography of the Atlantic island laurel forests. Journal of Biogeography 38: 226-246.
