# Supplementary material for: Biogeography of Mediterranean Hotspot Biodiversity: Re-Evaluating the 'Tertiary Relict' Hypothesis of Macaronesian Laurel Forests
Source: PLoS One. 2015 Jul 14;10(7):e0132091. doi: 10.1371/journal.pone.0132091 (PMC4501571; doi:10.1371/journal.pone.0132091)
Supplement: S2 Table — (PDF) [file pone.0132091.s002.pdf]

S2 Table. Genera of Macaronesian laurel forest plants with their distribution on the archipelagos (own data; [1]).

**Genus**	**Canaries**	**Madeira**	**Azores**
Adenocarpus	X	X	
Aeonium	X		
Aichryson	X	X	X
Andryala	X	X	
Apollonias	X	X	
Arbutus	X		
Arceuthobium			X
Argyranthemum	X	X	
Asparagus	X	X	
Asplenium	X	X	X
Athyrium	X	X	
Bencomia	X		
Blechnum	X	X	X
Bystropogon	X	X	
Canarina	X		
Carduus	X	X	
Carex	X	X	X
Cedronella	X	X	
Chamaecytisus	X		
Cheirolophus	X	X	
Cirsium		X	
Clethra		X	
Convolvulus	X	X	
Crambe	X	X	
Cryptotaenia	X		

**Genus**	**Canaries**	**Madeira**	**Azores**
Juniperus	X	X	X
Lactuca			X
Laurus	X	X	X
Leontodon			X
Luzula	X	X	X
Lysimachia			X
Marcetella	X	X	
Maytenus	X	X	
Melanoselium		X	
Mercurialis	X		
Micromeria	X	X	
Monanthes	X	X	
Morella	X	X	X
Musschia		X	
Myrsine			X
Ocotea	X	X	
Oreopteris		X	
Pericallis (*Senecio*)	X	X	X
Persea	X	X	
Phyllis	X	X	
Picconia	X	X	X
Pittosporum		X	
Platanthera			X
Pleiomeris	X		
Polypodium	X	X	X

**Genus**	**Canaries**	**Madeira**	**Azores**
Culcita	X	X	X
Dactylorhiza		X	
Davallia	X	X	
Diplazium	X	X	X
Dorycnium	X		
Dracunculus	X	X	
Dryopteris	X	X	X
Elaphoglossum		X	X
Erica	X	X	X
Erysimum	X	X	
Euphorbia	X	X	X
Ferula	X		
Festuca	X	X	X
Frangula		X	X
Geranium	X	X	
Gesnouinia	X		
Goodyera		X	
Heberdenia	X	X	
Hedera	X	X	X
Helichrysum	X	X	
Hymenophyllum	X	X	X
Hypericum	X	X	X
Ilex	X	X	X
Isoplexis	X	X	
Ixanthus	X		

**Genus**	**Canaries**	**Madeira**	**Azores**
Polystichum	X	X	
Prunus	X	X	
Pteris	X	X	
Ranunculus	X	X	
Rhamnus	X		
Rubus	X	X	
Rubia	X	X	
Salix	X		
Sambucus	X		
Sanicula		X	
Scrophularia	X		
Semele	X		
Sibhtorpia			
Sideritis	X		
Smilax	X	X	
Solanum	X		
Sonchus	X		
Teline	X		
Teucrium	X		
Trichomanes	X	X	
Vaccinium		X	
Viburnum	X	X	
Viola	X		
Visnea	X		
Woodwardia	X	X	

**Summary**		
**Sets**	**Code**	**Number of genera**
Canaries	A	81
Madeira	B	80
Azores	C	41
Canaries only	A\(BUC)	12
Madeira only	B\(AUC)	9
Azores only	C\(AUB)	7
Canaries and Madeira	A∩B	38
Canaries and Azores	A∩C	1
Madeira and Azores	B∩C	3
Canaries and Madeira and Azores	A∩B∩C	30

1. Acebes-Ginovés JR, León Arencibia MC, Rodríguez-Navarro ML, del Arco Aguilar M, García Gallo A, et al. (2010) Pteridophyta, Spermatophyta. In: Arechavaleta M, Rodríguez S, Zurita N, García A, editors. Lista de especies silvestres de Canarias Hongos, plantas y animales terrestres: Gobierno de Canarias. pp. 119-172.
