# Supplementary material for: Biogeography of Mediterranean Hotspot Biodiversity: Re-Evaluating the 'Tertiary Relict' Hypothesis of Macaronesian Laurel Forests
Source: PLoS One. 2015 Jul 14;10(7):e0132091. doi: 10.1371/journal.pone.0132091 (PMC4501571; doi:10.1371/journal.pone.0132091)
Supplement: S3 Table — (PDF) [file pone.0132091.s003.pdf]

S3 Table. Fossil record of selected genera representing laurophyllous vegetation of Europe and Macaronesia from the Neogene and Paleogene.

**Age**	**Region**	**Locality**	**Laurophyllous taxa**	**Reference**
Early Eocene	Western Europe	London Clay, Great Britain	*Beilschmiedia, Cinnamomum, Litsea*	[1]
Eocene, Oligocene, Miocene	Central Europe	Several locations in Germany, Czech Rep.	*Polyspora, Ternstroemites*	[2]
Oligocene	Central Europe	Several locations in Germany, Czech Rep., Slovenia	*Ilex*	[3]
Middle Eocene	Central Europe	Messel, Germany	*Camelliacarpoidea, Daphnogene, Laurocarpum, Laurophyllum*	[4] [5]
Late Eocene	Central Europe	Weisselster Basin, Germany	*Daphnogene, Laurophyllum*	[6]
Eocene/Oligocene transition	Central Europe	Roudníky area, Czech republic	*Laurophyllum*	[7]
Early Oligocene	Central Europe	Eger, Hungary	*Laurophyllum*	[8]
Middle Oligocene	Central Europe	Flörsheim, Germany	*Daphnogene, Gordonia, Laurophyllum, Laurus, Myrica, Ternstroemites*	[9]
Middle Oligocene	Central Europe	Calau Beds, Germany	*Laurocarpum, Phoebe, Stewartia, Visnea*	[10]
Late Oligocene	Central Europe	Lusatia, Germany	*Eurya, Laurocarpum, Ocotea, Ternstroemia*	[11]
Late Oligocene	Central Europe	Linz, Austria	*Daphnogene, Laurophyllum, Myrica*	[12]
Early Miocene, ~ 19-17 m.a.	Central Europe	Oberdorf, Austria	*Cinnamomum, Cleyera, Eurya, Gordonia, Ilex, Laurophyllum, Laurus, Litsea, Myrica, Ternstroemia*	[13,14] [15]
Early Miocene	Southern Europe	Aliveri, Greece	*Daphnogene, Laurophyllum, Myrica*	[16]

Early Miocene	Central Europe	Hrádek nad Nisou (Neisse), Czech Rep.	*Cinnamomum*, *Eurya*, *Ilex*, *Laurophyllum*, *Laurus*, *Leucothoe*, *Myrica*, *Ocotea*, *Phoebe*, *Ternstroemia*	[17]
Early/Middle Miocene	Central Europe	Berzdorf, Germany	*Cinnamomum*, *Eurya*, *Ilex*, *Laurocarpum*, *Myrica*, *Ocotea*, *Ternstroemia*,	[18]
Early/Middle Miocene	Central Europe	Wackersdorf, Germany	*Cinnamomum*, *Eurya*, *Ilex*, *Laurophyllum*, *Laurus*, *Myrica*, *Ocotea*, *Persea*, *Phoebe*, *Ternstroemia*, *Visnea*	[19] [20]
Middle Miocene	Central Europe	Sopron-Piusz-puszta, Hungary	*Laurophyllum*	[21]
Middle Miocene	Central Europe	Soby; Fasterholt; Denmark	*Visnea*, *Eurya*	[22]
Middle/Late Miocene	Central Europe	Lusatia, Germany	*Cinnamomum*, *Eurya*, *Ilex*, *Stewartia*, *Ternstroemia*	[23]
Late Miocene	Central Europe	Mataschen, Austria	*Daphnogene*, *Eurya*, *Gordonia*, *Laurophyllum*, *Laurus*, *Myrica*, *Schima*	[24] [25]
Late Miocene	Central Europe	Hambach, Germany	*Cinnamomum*, *Eurya*, *Ilex*, *Laurophyllum*, *Myrica*	[26] [27]
Early Late Miocene	Eastern Europe	Valea de Cris, Romania	*Daphnogene*, *Laurophyllum*	[28]
Late Miocene (Pontian/Messinian)	Southern Europe	Vegora, Greece	*Daphnogene*, *Laurophyllum*	[29]
Pliocene	Central Europe	Hambach, Germany	*Myrica*, *Ilex*, *Stewartia*	[30]
Pliocene	Macaronesia	Gran Canaria	cf. Arbutus, cf. Hedera, cf. Ilex, cf. Lauraceae genera	[31]
Latest Early Pliocene and/or early Middle Pliocene	Southern Europe	Asti, Italy	*Daphnogene*, cf. *Laurophyllum*, *Ocotea*	[32] [4]

Late Pliocene/Early Quaternary (Villafranchium)	Southern Europe	Rio Ca' Viettone, Italy	*Cinnamomum*, *Erica*, *Eurya*, *Ilex*, *Lindera*, *Ocotea*, *Phoebe*, *Stewartia*, *Ternstroemia*, *Visnea*	[25] [33] [34]
Pliocene or Pleistocene	Macaronesia	St. Jorge, Madeira	*Clethra*, *Erica*, *Laurus*, *Vaccinium*, *Ocotea*, *Woodwardia*	[31] [35]

1. Collinson ME (1983) Fossil plants of the London Clay. Palaeontological Association Field Guides to Fossils 1: 1-121.
2. Kvaček Z, Walther H (1984) Nachweis tertiärer Theaceen Mitteleuropas nach blatt-epidermalen Untersuchungen. II. Teil - Bestimmung fossiler Theaceen-Sippen. Feddes Repertorium 95: 331-346.
3. Walther H, Kvaček Z (2008) Die Gattung *Ilex* L.(Aquifoliaceae) im Paläogen von Mitteleuropa. Feddes Repertorium 119: 172-190.
4. Wilde V (1989) Untersuchungen zur Systematik der Blattreste aus dem Mitteleozän der Grube Messel bei Darmstadt (Hessen, Bundesrepublik Deutschland): A systematic study of leaf remains from the Middle Eocene of Grube Messel near Darmstadt (Hessen, Federal Republic of Germany). Courier Forschungsinstitut Senckenberg 115: 1-213.
5. Collinson ME, Manchester SR, Wilde V (2012) Fossil fruits and seeds of the Middle Eocene Messel biota, Germany. Abhandlungen der Senckenberg Gesellschaft für Naturforschung 570: 1-251.
6. Kunzmann L, Walther H (2002) Eine obereozäne Blätterflora aus dem mitteldeutschen Weißelster-Becken. Paläontologische Zeitschrift 76: 261-282.
7. Kvaček Z, Teodoridis V, Mach K, Přikryl T, Dvořák Z (2014) Tracing the Eocene-Oligocene transition: a case study from North Bohemia. Bulletin of Geosciences 89: 21-66.
8. Kvaček Z, Hably L (1998) New fossil elements in the Tard Clay Formation from Eger-Kiseged. Acta Palaeobotanica 38: 5-23.
9. Kvaček Z (2004) Revisions to the Early Oligocene flora of Flörsheim (Mainz Basin, Germany) based on epidermal anatomy. Senckenbergiana Lethaea 84: 1-73.
10. Mai DH (1998) Contribution to the flora of the middle Oligocene Calau Beds in Brandenburg, Germany. Review of Palaeobotany and Palynology 101: 43-70.
11. Mai DH (1997) Die oberoligozänen Floren am Nordrand der sächsischen Lausitz. Palaeontographica Abteilung B: 1-124.
12. Kovar J (1982) Eine Blätter-Flora des Egerien (Ober-Oligozän) aus marinen Sedimenten der Zentralen Paratethys im Linzer Raum (Osterreich). Beiträge zur Paläontologie von Österreich 9: 1-209.
13. Kovar-Eder J, Meller B (2001) Plant assemblages from the hanging wall sequence of the opencast mine Oberdorf N Voitsberg, Styria (Austria, Early Miocene, Ottnangian). Palaeontographica Abteilung B 259: 65-112.
14. Meller B (1998) Systematisch-taxonomische Untersuchungen von Karpo-Taphocoenosen des Köflach-Voitsberger Braunkohlenrevieres (Steiermark, Österreich; Untermiozän) und ihre paläoökologische Bedeutung. Jahrbuch der Geologischen Bundesanstalt 140: 497-655.
15. Meller B, Kovar-Eder J, Zetter R (1999) Lower Miocene leaf, palynomorph, and diaspore assemblages from the base of the lignite-bearing sequence in the opencast mine Oberdorf, N Voitsberg (Styria, Austria) as an indication of Younger Mastixioid vegetation. Palaeontographica Abteilung B 252: 123-179.

16. Velitzelos E, Kvaček Z, Buzek C (1992) Contributions to the Lower Miocene flora of Aliveri (Island of Evia, Greece). Documenta naturae 74: 10-25.
17. Holý F, Kvaček Z, Teodoridis V (2012) A review of the early Miocene mastixioid flora of the Kristina Mine at Hrádek nad Nisou in North Bohemia (Czech Republic). Acta Musei Nationalis Pragae, Series B-Historia Naturalis 68.
18. Czaja A (2003) Paläokarpologische Untersuchungen von Taphozönosen des unter-und mittelmiozäns aus dem Braunkohlentagebau Berzdorf/Oberlausitz (Sachsen). Palaeontographica Abteilung B 265: 1-148.
19. Knobloch E, Kvaček Z (1976) Miozäne Blätterfloren vom Westrand der Böhmischen Masse. Rozpravy Ustredniho Ustavu Geologickeho 42: 5-129.
20. Gregor H-J (1978) Die mittelmiozänen Frucht- und Samenfloren der Oberpfälzer Braunkohle. I. Funde aus den sandigen Zwischenmitteln. Palaeontographica Abteilung B 167: 8-103.
21. Boglárka E (1996) Sopron-Piusz-puszta szarmata makroflórája. In: Hably L, editor. Emlékkötet Andreánszky Gábor Születésének 100 Évfordulójára. Budapest Magyar Természettudományi Múzeum (Studia Naturalia, 9). pp. 83 - 98.
22. Friis EM (1979) The Damgaard flora: a new Middle Miocene flora from Denmark. Bulletin of the Geological Society Denmark 27: 117-142.
23. Mai DH (2001) Die mittelmiozänen und obermiozänen Floren aus der Meuroer und Raunoer Folge in der Lausitz. Teil II: Dicotyledones. Palaeontographica Abteilung B 257: 35-174.
24. Kovar-Eder J, Hably L (2006) The flora of Mataschen-a unique plant assemblage from the late Miocene of eastern Styria (Austria). Acta Palaeobotanica 46: 157-233.
25. Meller B, Hofmann CC (2004) Paleoecology of Diaspore- and Palynomorph assemblages form Late Miocene lake sediments (Mataschen near Fehring, East Styria, Austria). Joannea Geologie und Paläontologie 5: 177-217.
26. van Stroe M (1996) The flora of the Miocene 7b1 layer of Hambach, Germany. Documenta Naturae 104: 1-18.
27. Burgh Jvd (1983) Allochthonous Seed and Fruit Floras from the Pliocene of the Lower Rhine Basin. Review of Palaeobotany and Palynology 40: 33-90.
28. Givulescu R (1975) Fossile Pflanzen aus dem unteren Pannon Valea de Cris (Kreis Bihor, Rumänien). Acta Palaeobotanica 16: 71-82.
29. Kvaček Z, Velitzelos D, Velitzelos E (2002) Late Miocene flora of Vegora Macedonia N Greece. Athens: Panepistimiopolis. 175 p.
30. Burgh Jvd (1987) Miocene floras in the lower Rhenish Basin and their ecological interpretation. Review of Palaeobotany and Palynology 52: 299-366.
31. Anderson CL, Channing A, Zamuner AB (2009) Life, death and fossilization on Gran Canaria–implications for Macaronesian biogeography and molecular dating. Journal of Biogeography 36: 2189-2201.
32. Martinetto E (2003) Leaves of terrestrial plants from the Pliocene shallow marine and transitional deposits of Asti (Piedmont, NW Italy). Bollettino-Societa Paleontologica Italiana 42: 75-114.
33. Bertoldi R, Martinetto E (1995) Ricerche paleobotaniche (palinologiche e paleocarpologiche) sulla successione Villafranchiana del Rio Ca'Viettone (Torino, Italia). Il Quaternario 8: 403-422.
34. Martinetto E (1995) Significato cronologico e paleoambientale dei macrofossili vegetali nell'inquadramento stratigrafico del'Villafranchiano'di alcuni settori del Piemonte (Italia NW). Torino: Dipartimento di Sci. della Terra, Univ. degli Studi di Torino. 149 p.
35. Heer O (1857) Über die fossilen Pflanzen von St. Jorge in Madeira. Neue Denkschriften der Allgemeinen Schweizerischen Gesellschaft für die Gesammten Naturwissenschaften 15: 1-40.
