# Supplementary material for: Biogeography of Mediterranean Hotspot Biodiversity: Re-Evaluating the 'Tertiary Relict' Hypothesis of Macaronesian Laurel Forests
Source: PLoS One. 2015 Jul 14;10(7):e0132091. doi: 10.1371/journal.pone.0132091 (PMC4501571; doi:10.1371/journal.pone.0132091)
Supplement: S4 Table — In the second column applied ages and their source are stated. m: mean; s: standard deviation; mn: minimal age; mx: maximum age; loHPD: lowest value of 95% HPD. (PDF) [file pone.0132091.s004.pdf]

S4 Table. Calibration points and variable parameters used for the BEAST analyses. In the second column applied ages and their source are stated. m: mean; s: standard deviation; mn: minimal age; mx: maximum age; loHPD: lowest value of the 95% HPD.

**Phylogeny of**	**Used ages for calibration**	**Data set**	**Prior distribution**	**Prior parameters**	**2.5/median/97.5 % quantiles**	**Calibrated node**	**Reference**
Sambucus nigra ssp. *palmensis, Viburnum rigidum* (Adoxaceae)	Published: m=70.59; loHPD=49.73	nrITS	normal	mean=70.59 stdev=10.65	49.72/91.46	crown node Adoxaceae	[1]
Aeonium cuneatum, Aichryson pachycaulon group (Crassulaceae)	Published: m=41, 39, 41, 47, 50; loHPD=36	matK	normal	mean=44.42 stdev=4.3	35.99/52.85	crown node of Crassulaceae	[2,3]
	outcome of matK analysis: m=6.73; s=1.50	nrITS	normal	mean=6.73 stdev=1.50	3.79/9.67	crown node of *Aichryson-Monanthes-Aeonium*	
	outcome of matK analysis: m=9.63; s=2.07	nrITS	normal	mean=9.63 stdev=2.07	5.57/13.69	stem node of *Aichryson-Monanthes-Aeonium*	
Euphorbia mellifera/ stygiana (Euphorbiaceae)	Fossil: mn=37.2; mx=90	nrITS	lognormal	log(mean)=1.0 log(stdev)=1.51 offset=37.2	37.34/89.63	stem node Hippomaneae	[4,5]
	Published: mn=101.1; mx=119.4	nrITS	normal	mean=110.25 stdev=4.66	101.1-119.4	crown node of Malpighiales	[6,7]
Ixanthus viscosus (Gentianaceae)	Published: m=46, 52, 52, 50, 53; loHPD=37	matK, nrITS	normal	mean=50.75 stdev=7.00	37.03/64.47	stem node of Gentianaceae	[2,3]
Laurus novocanariensis/ azorica, *Ocotea foetens*,	Fossil: mn=106.8; mx=133	matK	lognormal	log(mean)=1.3 log(stdev)=1.0 offset=106.4	106.9/132.4	stem node of Lauraceae	[3,8]

Phylogeny of	Used ages for calibration	Data set	Prior distribution	Prior parameters	2.5/median/97.5 % quantiles	Calibrated node	Reference
Persea indica, Apollonias barbujana (Lauraceae)							
Picconia excelsa/ azorica (Oleaceae)	Published settings: m=40.0; s=3.0	nrITS	normal distribution	mean=40.0 stdev=3.0	34.12/40.0/45.8 8	crown node Oleeae+*Fraxinus*	[9]
Heberdenia excelsa, Pleiomeris canariensis (Primulaceae)	Fossil: mn=5.3; mx=23	nrITS	lognormal	log(mean)=1.0 log(stdev)=1.33 1 offset=5.1	5.3/42.02	stem node of *Androsace*	[10]
	Published: m=26, 33, 31, 40, 44; loHPD=30	nrITS	normal	mean=35.25 stdev=2.68	30.0/40.5	stem node of Primulaceae	[2,3]
Rhamnus glandulosa (Rhamnaceae)	Published settings: m=27.6 28.5; s=2.7	nrITS	normal	mean=28.05 stdev=2.7	22.76/28.05/33. 34	crown node Rhamneae	[11]
Prunus lusitanica (Rosaceae)	Fossil: mn=47.8; mx=77.4	nrITS	lognormal	log(mean)=0.0 log(stdev)=1.72 9 offset=47.78	48.0/77.37	stem node *Prunus*	[12,13]
Arbutus canariensis (Ericaceae)	Fossil: mn=11.8; mx=23	nrITS	lognormal	log(mean)=0.0 log(stdev)=1.23 2 offset=11.71	11.8/22.9	stem node American-mediterranean Arbitoideae	[14]
	Published: m=12,14,14,12,15; loHPD=7	nrITS	normal	mean=14.09 stdev=4.31	5.0643/ 14.09/22.54	Stem node European *Arbutus* and *Arctostaphylos*	[2,3]
Isoplexis group (Plantaginaceae)	Published: m=21, 25, 31, 24, 25; loHPD=17	nrITS	normal	mean=25.08 stdev=4.1	17.04/33.12	crown node *Plantago*+*Digitalis*	[2,3]
Bystropogon sect. *Canariense* (Lamiaceae)	Published (Appendix S3):	nrITS, trnL-F	normal	mean=10.0 stdev=1.54	6.98/13.02	crown node *Ziziphora*+*Bystrop ogon*	[15]

Phylogeny of	Used ages for calibration	Data set	Prior distribution	Prior parameters	2.5/median/97.5 % quantiles	Calibrated node	Reference
	m=10.00; loHPD=6.99						

1. Bell CD, Donoghue MJ (2005) Dating the Dipsacales: comparing models, genes, and evolutionary implications. American Journal of Botany 92: 284-296.
2. Wikström N, Savolainen V, Chase MW (2001) Evolution of the angiosperms: calibrating the family tree. Proceedings of the Royal Society of London Series B: Biological Sciences 268: 2211-2220.
3. Bell CD, Soltis DE, Soltis PS (2010) The age and diversification of the angiosperms re-revisited. American Journal of Botany 97: 1296-1303.
4. Bruyns PV, Klak C, Hanáček P (2011) Age and diversity in Old World succulent species of Euphorbia (Euphorbiaceae). Taxon 60: 1717-1733.
5. Zecca G, Casazza G, Minuto L, Labra M, Grassi F (2011) Allopatric divergence and secondary contacts in Euphorbia spinosa L: Influence of climatic changes on the split of the species. Organisms Diversity and Evolution 11: 357-372.
6. Dilcher LD, Manchester SR (1988) Investigations of angiosperms from the Eocene of North America: a fruit belonging to the Euphorbiaceae. Tertiary Research 9: 45.
7. Davis CC, Webb CO, Wurdack KJ, Jaramillo CA, Donoghue MJ (2005) Explosive radiation of Malpighiales supports a mid- Cretaceous origin of modern tropical rain forests. The American Naturalist 165: E36-E65.
8. von Balthazar M, Pedersen KR, Crane PR, Stampanoni M, Friis EM (2007) *Potomacanthus lobatus* gen. et sp. nov., a new flower of probable Lauraceae from the Early Cretaceous (Early to Middle Albian) of eastern North America. American Journal of Botany 94: 2041-2053.
9. Besnard G, Rubio de Casas R, Christin P-A, Vargas P (2009) Phylogenetics of *Olea* (Oleaceae) based on plastid and nuclear ribosomal DNA sequences: Tertiary climatic shifts and lineage differentiation times. Annals of Botany 104: 143-160.
10. Dorofeev PI (1963) Primulaceae. In: Orlov J, editor. Basics of paleontology: Gymnosperms, Angisperms. Moskva-Leningrad: Nauka. pp. 517-518.
11. Richardson JE, Chatrou LW, Mols JB, Erkens RHJ, Pirie MD (2004) Historical biogeography of two cosmopolitan families of flowering plants: Annonaceae and Rhamnaceae. Philosophical Transactions of the Royal Society of London Series B: Biological Sciences 359: 1495-1508.
12. Li Y, Smith T, Liu C-J, Awasthi N, Yang J, et al. (2011) Endocarps of *Prunus* (Rosaceae: Prunoideae) from the early Eocene of Wutu, Shandong Province, China. Taxon 60: 555-564.
13. Chin S-W, Shaw J, Haberle R, Wen J, Potter D (2014) Diversification of almonds, peaches, plums and cherries–Molecular systematics and biogeographic history of *Prunus* (Rosaceae). Molecular phylogenetics and evolution 76: 34-48.
14. Wolfe JA (1964) Miocene floras from Fingerrock Washington, southwestern Nevada. US Geological Survey Professional Paper: 1.
15. Drew BT, Sytsma KJ (2012) Phylogenetics, biogeography, and staminal evolution in the tribe Mentheae (Lamiaceae). American Journal of Botany 99: 933-953.
