# Supplementary material for: Biogeography of Mediterranean Hotspot Biodiversity: Re-Evaluating the 'Tertiary Relict' Hypothesis of Macaronesian Laurel Forests
Source: PLoS One. 2015 Jul 14;10(7):e0132091. doi: 10.1371/journal.pone.0132091 (PMC4501571; doi:10.1371/journal.pone.0132091)
Supplement: S5 Table — (PDF) [file pone.0132091.s005.pdf]

S5 Table. Published phylogenies of the studied taxa.

**Taxon**	**Reference**
Aeonium cuneatum	[1,2]
Arbutus canariensis	[3]
Aichryson pachycaulon	[4]
Apollonias barbujana	[5], [6] [7], [8]
Bystropogon sect. *Canariense*	[9,10]
Euphorbia mellifera	[11], [12]
Heberdenia excelsa	[13], [14]
Ixanthus viscosus	[15], [16]
Isoplexis group	[17]
Laurus novocanariensis / L. azorica	[5], [7,18] [19]
Ocotea foetens	[5] [6,7]
Persea indica	[5] [7] [20] [8]
Picconia excelsa	[21] [22]
Pleiomeris canariensis	[14]
Prunus lusitanica	[23-26]
Rhamnus glandulosa	[26-29]
Sambucus nigra subsp. *palmensis*	[30]
Viburnum rigidum	[31-35]

1. Mort ME, Soltis DE, Soltis PS, Francisco-Ortega J, Santos-Guerra A (2002) Phylogenetics and evolution of the Macaronesian clade of Crassulaceae inferred from nuclear and chloroplast sequence data. Systematic Botany 27: 271-288.
2. Thiv M, Esfeld K, Koch M (2010) Studying Adaptive Radiation at the Molecular Level: A Case Study in the Macaronesian Crassulaceae-Sempervivoideae. In: Glaubrecht M, editor. Evolution in Action: Springer. pp. 35-59.
3. Hileman LC, Vasey MC, Thomas Parker V (2001) Phylogeny and biogeography of the Arbutoideae (Ericaceae): implications for the Madrean-Tethyan hypothesis. Systematic Botany 26: 131-143.
4. Fairfield K, Mort M, Santos-Guerra A (2004) Phylogenetics and evolution of the Macaronesian members of the genus Aichryson (Crassulaceae) inferred from nuclear and chloroplast sequence data. Plant Systematics and Evolution 248: 71-83.
5. Rohwer JG (2000) Toward a phylogenetic classification of the Lauraceae: evidence from matK sequences. Systematic Botany 25: 60-71.
6. Chanderbali AS, van der Werff H, Renner SS (2001) Phylogeny and historical biogeography of Lauraceae: evidence from the chloroplast and nuclear genomes. Annals of the Missouri Botanical Garden: 104-134.
7. Rohwer JG, Rudolph B (2005) Jumping genera: the phylogenetic positions of *Cassytha*, *Hypodaphnis*, and *Neocinnamomum* (Lauraceae) based on different analyses of trnK intron sequences. Annals of the Missouri Botanical Garden 92: 153-178.

8. Li L, Li J, Rohwer JG, van der Werff H, Wang Z-H, et al. (2011) Molecular phylogenetic analysis of the Persea group (Lauraceae) and its biogeographic implications on the evolution of tropical and subtropical Amphi-Pacific disjunctions. American journal of botany 98: 1520-1536.
9. Trusty JL, Olmstead RG, Bogler DJ, Santos-Guerra A, Francisco-Ortega J (2004) Using molecular data to test a biogeographic connection of the Macaronesian genus *Bystropogon* (Lamiaceae) to the New World: a case of conflicting phylogenies. Systematic botany 29: 702-715.
10. Trusty JL, Olmstead RG, Santos-Guerra A, Sa-Fontinha S, Francisco-Ortega J (2005) Molecular phylogenetics of the Macaronesian-endemic genus Bystropogon (Lamiaceae): palaeo-islands, ecological shifts and interisland colonizations. Molecular Ecology 14: 1177-1189.
11. Molero J, Garnatje T, Rovira A, Garcia-Jacas N, Susanna A (2002) Karyological evolution and molecular phylogeny in Macaronesian dendroid spurges (*Euphorbia* subsect. *Pachycladae*). Plant Systematics and Evolution 231: 109-132.
12. Barres L, Vilatersana R, Molero J, Susanna A, Galbany-Casals M (2011) Molecular phylogeny of *Euphorbia* subg. *Esula* sect. *Aphyllis* (Euphorbiaceae) inferred from nrDNA and cpDNA markers with biogeographic insights. Taxon 60: 705-720.
13. Stähl B (1996) The relationships of *Heberdenia bahamensis* and *H. penduliflora* (Myrsinaceae). Botanical Journal of the Linnean Society 122: 315-333.
14. Martins L, Oberprieler C, Hellwig F (2003) A phylogenetic analysis of Primulaceae sl based on internal transcribed spacer (ITS) DNA sequence data. Plant Systematics and Evolution 237: 75-85.
15. Thiv M, Struwe L, Kadereit JW (1999) The phylogenetic relationships and evolution of the Canarian laurel forest endemic *Ixanthus viscosus* (Aiton) Griseb. (Gentianaceae): Evidence from matK and ITS sequences, and floral morphology and anatomy. Plant Systematics and Evolution 218: 299-317.
16. Mansion G, Zeltner L, Bretagnolle F (2005) Phylogenetic patterns and polyploid evolution within the Mediterranean genus Centaurium (Gentianaceae-Chironieae). Taxon 54: 931-950.
17. Bräuchler C, Meimberg H, Heubl G (2004) Molecular phylogeny of the genera Digitalis L. and Isoplexis (Lindley) Loudon (Veronicaceae) based on ITS-and trnL-F sequences. Plant Systematics and Evolution 248: 111-128.
18. Arroyo-García R, Martínez-Zapater JM, Prieto JF, Álvarez-Arbesú R (2001) AFLP evaluation of genetic similarity among laurel populations (*Laurus* L.). Euphytica 122: 155-164.
19. Rodríguez- Sánchez F, Guzmán B, Valido A, Vargas P, Arroyo J (2009) Late Neogene history of the laurel tree (Laurus L., Lauraceae) based on phylogeographical analyses of Mediterranean and Macaronesian populations. Journal of Biogeography 36: 1270-1281.
20. Rohwer JG, Li J, Rudolph B, Schmidt SA, van der Werff H, et al. (2009) Is Persea (Lauraceae) monophyletic? Evidence from nuclear ribosomal ITS sequences. Taxon 58: 1153-1167.
21. Wallander E, Albert VA (2000) Phylogeny and classification of Oleaceae based on rps16 and trnL-F sequence data. American Journal of Botany 87: 1827-1841.
22. Besnard G, Rubio de Casas R, Christin P-A, Vargas P (2009) Phylogenetics of *Olea* (Oleaceae) based on plastid and nuclear ribosomal DNA sequences: Tertiary climatic shifts and lineage differentiation times. Annals of Botany 104: 143-160.
23. Chin S-W, Shaw J, Haberle R, Wen J, Potter D (2014) Diversification of almonds, peaches, plums and cherries–Molecular systematics and biogeographic history of *Prunus* (Rosaceae). Molecular phylogenetics and evolution 76: 34-48.
24. Potter D, Eriksson T, Evans RC, Oh S, Smedmark J, et al. (2007) Phylogeny and classification of Rosaceae. Plant systematics and evolution 266: 5-43.
25. Liu X-L, Wen J, Nie Z-L, Johnson G, Liang Z-S, et al. (2013) Polyphyly of the Padus group of Prunus (Rosaceae) and the evolution of biogeographic disjunctions between eastern Asia and eastern North America. Journal of plant research 126: 351-361.
26. Wen J, Berggren ST, Lee C-H, Ickert-Bond S, Yi T-S, et al. (2008) Phylogenetic inferences in *Prunus* (Rosaceae) using chloroplast ndhF and nuclear ribosomal ITS sequences. Journal of Systematics and Evolution 46: 322-332.
27. Richardson JE, Fay MF, Cronk QC, Bowman D, Chase MW (2000) A phylogenetic analysis of Rhamnaceae using rbcL and trnL-F plastid DNA sequences. American Journal of Botany 87: 1309-1324.

28. Richardson JE, Chatrou LW, Mols JB, Erkens RHJ, Pirie MD (2004) Historical biogeography of two cosmopolitan families of flowering plants: Annonaceae and Rhamnaceae. Philosophical Transactions of the Royal Society of London Series B: Biological Sciences 359: 1495-1508.
29. Holmgren K, Oxelman B (2004) Generic limits in *Rhamnus* L. sl (Rhamnaceae) inferred from nuclear and chloroplast DNA sequence phylogenies. Taxon: 383-390.
30. Eriksson T, Donoghue MJ (1997) Phylogenetic relationships of Sambucus and Adoxa (Adoxoideae, Adoxaceae) based on nuclear ribosomal ITS sequences and preliminary morphological data. Systematic Botany: 555-573.
31. Winkworth RC, Donoghue MJ (2005) Viburnum phylogeny based on combined molecular data: implications for taxonomy and biogeography. American Journal of Botany 92: 653-666.
32. Bell CD, Edwards EJ, Kim S-T, Donoghue MJ (2001) Dipsacales phylogeny based on chloroplast DNA sequences. Harvard Papers in Botany 6: 481-500.
33. Bell CD, Donoghue MJ (2005) Dating the Dipsacales: comparing models, genes, and evolutionary implications. American Journal of Botany 92: 284-296.
34. Moore BR, Donoghue MJ (2009) A Bayesian approach for evaluating the impact of historical events on rates of diversification. Proceedings of the National Academy of Sciences, USA 106: 4307-4312.
35. Desurmont GA, Donoghue MJ, Clement WL, Agrawal AA (2011) Evolutionary history predicts plant defense against an invasive pest. Proceedings of the National Academy of Sciences, USA 108: 7070-7074.
