# Supplementary material for: Biogeography of Mediterranean Hotspot Biodiversity: Re-Evaluating the 'Tertiary Relict' Hypothesis of Macaronesian Laurel Forests
Source: PLoS One. 2015 Jul 14;10(7):e0132091. doi: 10.1371/journal.pone.0132091 (PMC4501571; doi:10.1371/journal.pone.0132091)
Supplement: S6 Table — New sequences for this study are marked in italics. (PDF) [file pone.0132091.s006.pdf]

S6 Table. Taxa used for molecular analyses and according Genbank accession numbers. New sequences for this study are marked in italics.

Taxon sampling *Viburnum rigidum* and *Sambucus palmensis* (Adoxaceae)

**Taxon**	**nrITS**
Abelia chinensis R. Br.	FJ745388
Acanthocalyx nepalensis (D. Don) M. J. Cannon	AY290015
Adoxa moschatellina L.	U88194
Bupleurum longiradiatum Turcz.	JF837167
Bupleurum yunnanense Franch.	HQ687968
Centranthus lecoqii Jord.	DQ354165
Cryptothladia chinensis (Y. Y. Pai) M. J. Cannon	AY236184
Cryptothladia chlorantha (Diels) M. J. Cannon	AY290012
Diervilla sessilifolia Buckley	AY236177
Dipelta yunnanensis Franch.	AY236180
Dipsacus asperoides C. Y. Cheng & Ai	EU925567
Fedia pallescens Mathez	AY310450
Hedera nepalensis K. Koch	GU054637
Heptacodium miconioides Rehder	EU240665
Kolkwitzia amabilis Graebn.	EU240666
Leycesteria formosa Wall.	AF265276
Lonicera caerulea L.	EU240677
Morina longifolia Wall. ex DC.	AY236185
Plectritis brachystemon Fisch. & C. A. Mey.	AY792828
Pterocephalus papposus Coult.	FJ640773
Sambucus adnata Wall ex DC.	JF978509
Sambucus australis Cham. & Schltdl.	U88196
Sambucus ebulus L.	DQ679819
Sambucus javanica Blume	U88201
Sambucus nigra L.	DQ521258
Sambucus nigra L.	HQ714455
Sambucus nigra subsp. *cerulea* (Raf.) Bolli	U88197
Sambucus nigra subsp. *maderensis* (Lowe) Bolli	*KJ188948*
Sambucus nigra subsp. *maderensis* (Lowe) Bolli	*KJ188949*

Taxon	nrITS
Sambucus nigra subsp. *palmensis* (Link) Bolli	*KJ188945*
Sambucus nigra subsp. *palmensis* (Link) Bolli	*KJ188946*
Sambucus nigra subsp. *palmensis* (Link) Bolli	*KJ188947*
Sambucus nigra subsp. *palmensis* (Link) Bolli	*KJ188950*
Sambucus nigra subsp. *peruviana* (Kunth) Bolli	U88205
Sambucus racemosa L.	HQ714459
Sambucus wightiana Wall.	U88208
Sambucus williamsii Hance	AF366929
Sinadoxa corydalifolia C. Y. Wu, Z. L. Wu & R. F. Huang	AF248611
Symphoricarpos sinensis Rehder	GQ268148
Triosteum himalayanum Wall.	AF265286
Triplostegia glandulifera Wall. ex DC.	AY236189
Valeriana officinalis L. & Maillefer	DQ180745
Valeriana tuberosa L.	DQ354166
Viburnum acerifolium L.	AY265114
Viburnum cinnamomifolium Rehder	AY265116
Viburnum clemensae J. Kern	AY265117
Viburnum davidii Franch.	AY265120
Viburnum furcatum Blume ex Hook. f. & Thomson	AB237444
Viburnum japonicum Spreng.	AY265131
Viburnum jucundum C. V. Morton	AY265132
Viburnum lantana L.	FJ490781
Viburnum opulus L.	AF366928
Viburnum rigidum Vent.	*KJ188951*
Viburnum rigidum Vent.	*KJ188952*
Viburnum rigidum Vent.	*KJ188953*
Viburnum rigidum Vent.	*KJ188954*
Viburnum rigidum Vent.	*KJ188955*
Viburnum rigidum Vent.	*KJ188956*
Viburnum rigidum Vent.	*KJ188957*
Viburnum rigidum Vent.	*KJ188958*
Viburnum rigidum Vent.	*KJ188959*
Viburnum tinus L.	*KJ188960*

Taxon	nrITS
Viburnum tinus L.	*KJ188961*
Viburnum tinus L.	*KJ188962*
Viburnum tinus L.	*KJ188963*
Viburnum tinus L.	*KJ188964*
Viburnum tinus L.	*KJ188965*
Viburnum tinus L.	JQ805228
Viburnum wrightii Miq.	HQ591986
Weigela florida A. DC.	AF078711

Taxon sampling *Aeonium cuneatum* and *Aichryson pachycaulon* (Crassulaceae)

Taxon	matK
Aeonium balsamiferum Webb & Berthel.	AY082161
Aeonium canariense Webb & Berthel.	AY082267
Aeonium castello-paivae Bolle	-
Aeonium ciliatum (Willd.) Webb & Berthel.	AY082163
Aeonium cuneatum Webb & Berthel.	-
Aeonium davidbramwellii H.-Y. Liu	AY082164
Aeonium decorum Webb ex Bolle	AY082165
Aeonium glandulosum (Aiton) Webb & Berthel.	AY082166
Aeonium glutinosum Webb & Berthel.	AY082167
Aeonium gomerense Praeger	-
Aeonium goochiae Webb & Berthel.	AF115579
Aeonium gorgoneum J. A. Schmidt	AY082169
Aeonium haworthii (Salm-Dyk) Webb & Berthel.	-
Aeonium hierrense (Murray) Pit. & Proust	-
Aeonium holochrysum Webb & Berthel.	AY082269
Aeonium korneliuslemsii H.-Y. Liu	AY082171
Aeonium lancerottense Praeger	-
Aeonium leucoblepharum Webb ex A. Rich.	AY082270
Aeonium lindleyi Webb & Berthel.	-
Aeonium mascaense Bramwell	AY082172
Aeonium nobile Praeger	AF115582
Aeonium palmense Webb ex Christ.	-
Aeonium percarneum (Murray) Pit. & Proust	AF115581
Aeonium percarneum (Murray) Pit. & Proust	-
Aeonium pseudourbicum Bañares	AY082177
Aeonium rubrolineatum Svent.	-
Aeonium saundersii Bolle	AY082175
Aeonium sedifolium (Webb ex Bolle) Pit. & Proust	AF115583

**Taxon**	**matK**
Aeonium simsii (Sweet) Stearn	AF115585
Aeonium smithii Webb & Berthel.	-
Aeonium spathulatum (Hornem.) Praeger	AF115584
Aeonium subplanum Praeger	AY082268
Aeonium tabuliforme (Haw.) Webb ex Berthel.	AF115577
Aeonium undulatum Webb & Berthel.	AF115580
Aeonium urbicum (C.Sm. ex Hornem.) Webb & Berthel.	AY082176
Aeonium vestitum Svent.	AF115586
Aeonium virgineum Webb ex Christ.	AF115578
Aeonium viscatum Webb ex Bolle	AY082271
Aeonium volkeri E.Hern. & Bañares	AY082162
Aichryson bethencourtianum Bolle	-
Aichryson divaricatum (Aiton) Praeger	-
Aichryson dumosum (Lowe) Praeger	-
Aichryson laxum (Haw.) Bramwell	AF115588
Aichryson pachycaulon Bolle	AY082157
Aichryson pachycaulon Bolle subsp. *gonzalez-hernandezii* (Kunk.) Bramwell	-
Aichryson pachycaulon Bolle subsp. *immaculatum* (Webb ex Christ) Bramwell	-
Aichryson pachycaulon Bolle subsp. *pachycaulon*	-
Aichryson pachycaulon Bolle subsp. *parviflorum* (Bolle) Bramwell	-
Aichryson pachycaulon Bolle subsp. *praetermisssum* Bramwell	-
Aichryson palmense Webb ex Bolle	AY082156
Aichryson parlatorei Bolle	-
Aichryson porphyrogennetos Bolle	-
Aichryson punctatum (Chr. Sm.) Webb ex Berthel.	AF115587
Aichryson tortuosum (Aiton) Webb & Berthel.	-
Aichryson villosum (Aiton) Webb & Berthel.	-
Cotyledon tomentosa Harv.	AF115592
Crassula deltoidea Thunb.	AF115599
Crassula fascicularis Lam.	AF115596
Crassula rupestris Thunb.	AF115602
Dudleya viscida (S.Watson) Moran	AF115604
Greenovia aizoon Bolle	
Greenovia aureum Webb & Berthel.	-
Greenovia aureum Webb & Berthel.	AF115614
Greenovia diplocycla Webb ex Bolle	AF115613
Greenovia dodrantalis Webb & Berthel.	AF115612
Kalanchoe scapigera Welw. ex Oliv.	AF115620
Lenophyllum acutifolium Rose	AF115625
Monanthes adenoscepes Svent.	AY082264
Monanthes amydros Svent.	AF115627
Monanthes anagensis Praeger	AF115626

Taxon	matK
Monanthes brachycaulon (Webb ex Bolle) Lowe	AY082265
Monanthes icterica (Webb ex Bolle) Praeger	AF115629
Monanthes laxiflora (DC.) Bolle	AY082266
Monanthes minima Bolle	AY082160
Monanthes polyphylla Haw.	AF115628
Monanthes subcrassicaulis (O. Ktze.) Praeger	AY082159
Orostachys fimbriata (Turcz.) A.Berger	AF115631
Penthorum sedoides L.	EF179063
Phedimus stoloniferus (S.G.Gmel.) 't Hart	AF115654
Pterostemon rotundifolius Ramirez	AF274630
Sedum burrito Moran	AF115655
Sedum caeruleum L.	-
Sedum dasyphyllum L.	AF115657
Sedum furfuraceum Moran	AF115659
Sedum fusiforme Lowe	AF115638
Sedum jaccardianum Maire & Wilczek	AF115637
Sedum jaccardianum Maire & Wilczek	-
Sedum laconicum Boiss. & Heldr.	AF115642
Sedum magellense Ten.	AF115644
Sedum modestum Ball	AF115639
Sedum modestum Ball	-
Sedum morrisonense Hayata	AF115651
Sedum oryzifolium Makino	AF115647
Sedum pubescens Vahl.	-
Sedum rupestre L.	AF115667
Sedum surculosum Coss.	-
Sempervivum tectorum L.	AF115671
Tetracarpaea tasmanica Hook. f.	EF179064
Tylecodon wallichii (Harv.) Toelken	AF115590
Umbilicus rupestris (Salisb.) Dandy	AF115684

Taxon sampling *Arbutus canariensis* (Ericaceae)

Taxon	nrITS
Arbutus andrachne L.	AF091954
Arbutus arizonica (A.Gray) Sarg.	AF091958
Arbutus canariensis Duhamel	AF091992; AF091993
Arbutus menziesii Pursh	AF086828
Arbutus occidentalis McVaugh & Rosatti	AF091955
Arbutus peninsularis Rose & Goldman	AF091956
Arbutus texana Buckley	AF091959; AF091960
Arbutus unedo L.	AF091952; AF091953

Taxon	nrITS
Arbutus xalapensis Kunth	AF091957
Arctostaphylos andersonii A.Gray	AF091991
Arctostaphylos hookeri G.Don	AF091951
Arctostaphylos manzanita Parry	AF352012
Arctostaphylos nummularia A.Gray	AF091949
Arctostaphylos pungens Kunth	AF106820
Arctostaphylos tomentosa (Pursh) Lindl.	AF106823
Arctostaphylos uva-ursi (L.) Spreng.	AF091950
Arctous alpina (L.) Nied.	AF091961
Arctous erythrocarpa Small	GQ281006
Arctous rubra (Rehder & E.H.Wilson) Nakai	AF091944
Comarostaphylis arbutoides Lindl.	KF419121
Comarostaphylis discolor (Hook.) Diggs	AF091945; AF091943
Comarostaphylis diversifolia (Parry) Greene	AF091947
Enkianthus campanulatus (Miq.) G.Nicholson	AF091940
Ornithostaphylos oppositifolia (Parry) Small	AF297794; AF091962
Pyrola rotundifolia L.	AF091939
Vaccinium ovatum Pursh	AY274568
Xylococcus bicolor Nutt.	AF091948

Taxon sampling *Euphorbia mellifera* (Euphorbiaceae)

Taxon	nrITS
Anthostema madagascariense Baill.	AF537582
Anthostema senegalense Juss.	JN250088
Calycopeplus casuarinoides L.S. Sm	AF537580
Croton alabamensis E.A.Sm. ex Chapm.	DQ227518
*Dichostemma glaucescens*L.	AF537584
Euphorbia abdelkuri Balf. f.	JN250102
Euphorbia angulata Jacq.	JN010025
Euphorbia angulata Jacq.	JN010026
Euphorbia antso Denis	AF537579
Euphorbia aphylla Brouss. ex Willd.	HQ900577
Euphorbia atropurpurea Brouss.	HQ900579
Euphorbia bourgaeana J.Gay ex Boiss.	JN250121
Euphorbia capitulata Rchb.	JN010032
Euphorbia capmanambatoensis Rauh	JQ952292
Euphorbia carniolica Jacq.	JN010033
Euphorbia cedrorum Rauh & Hebding	JN010033
*Euphorbia coniosperma*Boiss. & Buhse	HQ900578
Euphorbia conzattii V.W.Steinm.	GU214922
Euphorbia cymbifera (Schltdl.) V.W.Steinm.	GU214923
Euphorbia dentosa I.M.Johnst.	HQ645259

**Taxon**	**nrITS**
Euphorbia dimorphocaulon P.H.Davis	JN250137
Euphorbia dulcis L.	JN010041
Euphorbia eriantha Benth.	HQ645262
Euphorbia esula L.	HQ900604
Euphorbia falcata L.	JN010045
Euphorbia flavicoma DC.	JN250152
Euphorbia fragifera Jan	JN010048
Euphorbia georgei Oudejans	HQ645218
Euphorbia gymnocalycioides M.G.Gilbert & S.Carter	JN250165
Euphorbia gypsicola Rech.f. & Aellen	JF732976
Euphorbia hainanensis Croizat	JQ750922
Euphorbia helioscopia L.	EU659747
Euphorbia hofstaetteri Rauh	JQ952326
Euphorbia illirica Lam.	HQ900616
Euphorbia lagascae Spreng.	HQ900618
Euphorbia lamarckii Sweet	HQ900619
Euphorbia longituberculosa Hochst. ex Boiss.	JN250185
Euphorbia macroceras Fisch. & C.A.Mey.	JN010062
Euphorbia mainty Denis ex Leandri	DQ204870
Euphorbia mellifera Aiton	HQ900628
Euphorbia mellifera Aiton	*KJ189013*
Euphorbia mellifera Aiton	*KJ189014*
Euphorbia mellifera Aiton	*KJ189015*
Euphorbia mellifera Aiton	*KJ189016*
Euphorbia neoarborescens Bruyns	JN250195
Euphorbia nereidum Jahand. & Maire	JN250198
Euphorbia palustris L.	HQ900637
Euphorbia petrophila C.A.Mey.	GU984332
Euphorbia piscatoria Aiton	HQ900644
Euphorbia pithyusa L.	JN010079
Euphorbia platyclada Rauh	JN250213
Euphorbia platyphyllos L.	JN010081
Euphorbia polychroma A.Kern.	JN010082
Euphorbia pterococca Brot.	JN010085
Euphorbia regis-jubae J.Gay	JN250226
Euphorbia schimperiana Scheele	JN207816
Euphorbia sclerocyathium Korovin & Popov	JN250237
Euphorbia seibanica Lavranos & Gifri	JN207818
Euphorbia setiloba Engelm. ex Torr.	HQ645345
Euphorbia spinosa L.	HQ900656
Euphorbia stygiana subsps. santamariae H.Schäf.	HQ202071
Euphorbia stygiana H.C.Watson *subsp. stygiana*	HQ202078
Euphorbia sulcata Lens ex Loisel.	HQ900659
Euphorbia tannensis Spreng.	AF537425

Taxon	nrITS
Euphorbia tuckeyana Steud. ex Webb	KC212415
Euphorbia villosa Waldst. & Kit. ex Willd.	JN010100
Homalanthus nutans (G.Forst.) Guill.	JN250093
Homalanthus populifolius Graham	AF537585
Jatropha integerrima Jacq.	AY971261
Macaranga conifera (Rchb.f. & Zoll.) Müll.Arg.	DQ866544
Mallotus tetracoccus (Roxb.) Kurz	DQ866623
Manihot esculenta Crantz	GU214953
Moacroton ekmanii (Urb.) Croizat	DQ227528
Neoguillauminia cleopatra (Baill.) Croizat	JN250099
Pera glabrata (Schott) Poepp. ex Baill.	DQ787417
Stillingia spinulosa Torr.	AF537588
Stillingia sylvatica L.	JN250101

Taxon sampling *Ixanthus* (Gentianaceae)

Taxon	matK
Blackstonia acuminata (Koch & Ziz) Domin	-
Blackstonia imperfoliata (L. f.) Samp.	AJ010506; AJ011435
Blackstonia perfoliata (L.) Huds.	HM850819; HM850819
Canscora diffusa (Vahl) R. Br. ex Roem. & Schult.	AJ388143; AJ388212
Centaurium maritimum (L.) Fritsch	AJ010508; AJ011437
Chelonanthus alatus (Aubl.) Pulle	AJ010520; AJ011449
Cicendia filiformis (Lam.) Delabr.	AJ010510; AJ011439
Cinchona pubescens Vahl	Z70197
Coutoubea spicata Aubl.	AJ388150; AJ388219
Curtia tenuifolia (Aubl.) Knobl.	AJ388151; AJ388220
Deianira pallescens Cham. & Schlecht.	AJ388153; AJ388222
Duplipetala pentanthera (C. B. Clarke) Thiv	AJ010507; AJ011436
Enicostema axillare (Lam.) A.Raynal	AJ010513; AJ011442
Eustoma grandiflorum (Raf.) Shinners	AJ010514; AJ011443
Exacum tetragonum Roxb.	AJ388156; AJ388225
Fagraea elliptica Roxb.	AJ388158; AJ388227
Gardenia taitensis DC.	AJ388201; AJ388271
Gelsemium sempervirens (L.) Aiton	Z70195
Geniostemon gypsophilum B. L. Turner	AJ388161; AJ388231
Geniostoma rupestre Forst.	Z70194
Gentiana cruciata L.	AJ010519; AJ011448
Gentianella aspera (Heg. & Heer) Dostal ex Skalicky, Chrtek & Gill	AJ010517; AJ011446
Gentianopsis ciliata (L.) Ma	AJ388164; AJ388234
Ixanthus viscosus (Aiton) Griseb.	*KJ189063*

**Taxon**	**matK**
Ixanthus viscosus (Aiton) Griseb.	*KJ189060*
Ixanthus viscosus (Aiton) Griseb.	*KJ189059*
Ixanthus viscosus (Aiton) Griseb.	*KJ189062*
Ixanthus viscosus (Aiton) Griseb.	*KJ189057*
Ixanthus viscosus (Aiton) Griseb.	*KJ189061*
Ixanthus viscosus (Aiton) Griseb.	*KJ189058*
Jasminum fluminense Vell.	AJ388202; AJ388272
Lisianthius jefensis A. Robyns & T.S. Elias	AJ010522; AJ011451
Logania albiflora (Andrews & Jacks.) Druce	AJ388203; AJ388273
Macrocarpaea domingensis Urb. & Ekman	AJ010523; AJ011452
Microrphium pubescens C. B. Clarke	AJ388178; AJ388248
Ornichia madagascariensis (Baker) Klack.	AJ388182; AJ388252
Orphium frutescens (L.) E. Mey.	AJ010525; AJ011454
Sabatia angularis (L.) Pursh	AJ010526; AJ011455
Saccifolium bandeirae Maguire & Pires	AJ388187; AJ388257
Strumpfia maritima L.	AJ388204; AJ388274
Strychnos nux-vomica L.	Z70193
Swertia perennis L.	AJ010528; AJ011457
Symbolanthus calygonus (Ruiz & Pavon) Griseb.	AJ010529; AJ011458
Symphyllophyton caprifolioides Gilg	AJ010530; AJ011459
Tachia guianensis Aubl.	AJ011433; AJ011461
Tachiadenus carinatus (Desr.) Griseb.	AJ011434; AJ011460
Thevetia peruviana K. Schum.	Z70188
Voyriella parviflora (Miq.) Miq.	AJ388197; AJ388267

Taxon sampling *Apollonias barbujana*, *Laurus novocanariensis*, *Ocotea foetens*, *Persea indica* (Lauraceae)

**Taxon**	**matK**
Actinodaphne borneensis Meisn.	AJ247142
Aiouea dubia (HBK) Mez	AJ247143
Alseodaphne perakensis (Gamble) Kosterm.	AJ247144
Aniba affinis (Meisn.) Mez	AJ247145
Apollonias barbujana (Cav.) Bornm.	*KJ189033*
Apollonias barbujana (Cav.) Bornm.	*KJ189034*
Apollonias barbujana (Cav.) Bornm.	*KJ189035*
Apollonias barbujana (Cav.) Bornm.	*KJ189036*
Apollonias barbujana (Cav.) Bornm.	*KJ189037*
Apollonias barbujana (Cav.) Bornm.	*KJ189038*
Aspidostemon parvifolius (Scott-Elliot) van der Werff	AJ627912

**Taxon**	**matK**
Beilschmiedia berteroana (Gay) Kosterm.	AJ247147
Caryodaphnopsis tomentosa van der Werff	AJ627919
Chlorocardium rodiei (Schomb.) Rohwer, H.G.Richt. & van der Werff	AJ247153
Cinnamomum camphora (L.) J. Presl	AJ247154
Cryptocarya alba (Molina) Looser	AJ247158
Dehaasia cuneata (Blume) Blume	AJ247160
Dicypellium caryophyllaceum (Mart.) Nees	AJ247161
Endiandra pubens Meisn.	AJ247162
Endlicheria formosa A. C. Sm.	AJ247163
Eusideroxylon zwageri Teijsm. & Binn.	AJ627926
Hedycarya arborea J. R. Forst. & G. Forst.	AJ627927
Hernandia nymphaeifolia (J. Presl) Kubitzki	AJ247165
Hypodaphnis zenkeri (Engl.) Stapf	AJ247166
Iteadaphne caudata (Nees) H. W. Li	AF244408
Laurelia sempervirens Tul.	AJ627928
Laurus azorica (Seub.) Franco	HM850594
Laurus azorica (Seub.) Franco	FJ408918
Laurus azorica (Seub.) Franco	FJ408945
Laurus azorica (Seub.) Franco	*KJ189039*
Laurus nobilis L.	FJ408930
Laurus nobilis L.	FJ408871
Laurus nobilis L.	FJ408869
Laurus nobilis L.	FJ408890
Laurus nobilis L.	FJ408953
Laurus nobilis L.	FJ408881
Laurus novocanariensis Rivas Mart., Lousã, Fern.Prieto, E. Días, J. C. Costa & C. Aguiar	*KJ189041*
Laurus novocanariensis Rivas Mart., Lousã, Fern.Prieto, E. Días, J. C. Costa & C. Aguiar	*KJ189042*
Laurus novocanariensis Rivas Mart., Lousã, Fern.Prieto, E. Días, J. C. Costa & C. Aguiar	*KJ189043*
Laurus novocanariensis Rivas Mart., Lousã, Fern.Prieto, E. Días, J. C. Costa & C. Aguiar	*KJ189044*
Laurus novocanariensis Rivas Mart., Lousã, Fern.Prieto, E. Días, J. C. Costa & C. Aguiar	*KJ189045*
Laurus novocanariensis Rivas Mart., Lousã, Fern.Prieto, E. Días, J. C. Costa & C. Aguiar	*KJ189046*
Licaria triandra (Sw.) Kosterm.	AJ247168
Lindera benzoin (L.) Blume	AJ247169
Machilus grijsii Hance	AJ247180
Mezilaurus opaca Kubitzki & van der Werff	AJ247172
Nectandra riparia Rohwer	AJ247174

**Taxon**	**matK**
Neocinnamomum fargesii (Lecomte) Kosterm.	GU117743
Ocotea foetens (Aiton) Baill.	HM850596
Ocotea foetens (Aiton) Baill.	*KJ189047*
Ocotea foetens (Aiton) Baill.	*KJ189048*
Ocotea foetens (Aiton) Baill.	*KJ189049*
Ocotea foetens (Aiton) Baill.	*KJ189050*
Ocotea foetens (Aiton) Baill.	*KJ189051*
Ocotea malcomberi van der Werff	AJ627929
Ocotea paulii C. K. Allen	AJ247178
Ocotea veraguensis (Meisn.) Mez	JQ589824
Persea americana Mill.	AJ247179
Persea caerulea (Ruiz & Pav.) Mez	EU153875
Persea chamissonis Mez	JF966457
Persea cinerascens Blake	JF966452
Persea indica (L.) Spreng.	HM850597
Persea indica (L.) Spreng.	*KJ189052*
Persea indica (L.) Spreng.	*KJ189053*
Persea indica (L.) Spreng.	*KJ189054*
Persea indica (L.) Spreng.	*KJ189055*
Persea indica (L.) Spreng.	*KJ189056*
Persea lingue (Miers ex Bertero) Nees	AJ247182
Persea steyermarkii C. K. Allen	JF966439
Persea tolimanensis Zentmyer & Schieber	JF966468
Peumus boldus Molina	AJ247183
Phoebe formosana (Hayata) Hayata	AJ247184
Pleurothyrium costanense van der Werff	AJ247185
Potameia chartacea Kosterm.	AJ627930
Povedadaphne quadriporata W. C. Burger	AJ247186
Rhodostemonodaphne grandis (Mez) Rohwer	EU153879
Sassafras tzumu (Hemsl.) Hemsl.	AJ247188
Sextonia rubra (Mez) van der Werff	AJ627933
Sinosassafras flavinervia (C. K. Allen) H.W. Li	AF244390
Tambourissa religiosa (Tul.) A. DC.	AJ247189
Umbellularia californica (Hook. & Arn.) Nutt.	AJ247190
Urbanodendron verrucosum (Nees) Mez	AJ247145
Wilkiea hugeliana (Tul.) A. DC.	AJ627932
Williamodendron glaucophyllum (van der Werff) Kubitzki & H. G. Richt.	AJ247192

Taxon sampling *Picconia excelsa* (Oleaceae)

**Taxon**	**nrITS**
Chionanthus retusus (Lindl.) Paxton	AF231811
Forestiera acuminata (Michx.) Poir.	AF231819
Fraxinus americana L.	AF231825
Fraxinus excelsior L.	AF231830
Fraxinus quadrangulata Michx.	AF231833
Ligustrum vulgare L.	AF231848
Nestegis sandwicensis (A. Gray) O. Deg., I. Deg. & L. A. S. Johnson	AF231857
Norohnia emarginata (Lam.) Thouars	AF231858
Olea brachiata (Lour.) Merr.	AF231864
Olea europaea L.	AF231866
Olea maroccana Greuter & Burdet	*KJ188984*
Osmanthus americanus (L.) Benth. & Hook. f. ex A. Gray	AF231868
Osmanthus decorus Kasapligil	KJ188968
Osmanthus fragrans (Thunb.) Lour.	AF231869
Osmanthus heterophyllus (G. Don) P. S. Green	AF231870
Phillyrea angustifolia L.	*KJ188966*
Phillyrea angustifolia L.	*KJ188969*
Phillyrea latifolia L.	*KJ188967*
Phillyrea latifolia L.	*KJ188970*
Phillyrea latifolia L.	*KJ188971*
Phillyrea latifolia L.	AF231874
Picconia azorica (Tutin) Knobl.	*KJ188980*
Picconia azorica (Tutin) Knobl.	*KJ188981*
Picconia excelsa (Sol.) A. DC.	*KJ188972*
Picconia excelsa (Sol.) A. DC.	*KJ188973*
Picconia excelsa (Sol.) A. DC.	*KJ188974*
Picconia excelsa (Sol.) A. DC.	*KJ188975*
Picconia excelsa (Sol.) A. DC.	*KJ188976*
Picconia excelsa (Sol.) A. DC.	*KJ188977*
Picconia excelsa (Sol.) A. DC.	*KJ188978*
Picconia excelsa (Sol.) A. DC.	*KJ188979*
Picconia excelsa (Sol.) A. DC.	*KJ188982*
Picconia excelsa (Sol.) A. DC.	*KJ188983*
Syringa vulgaris L.	AF231882

Taxon sampling *Isoplexis canariensis*, *Isoplexis chalcantha*, *Isoplexis isabelliana* and *Isoplexis sceptrum* (Plantaginaceae)

**Taxon**	**nrITS**
Aragoa abietina Kunth	AJ459404
Aragoa cupressina Kunth	AJ459402
Digitalis atlantica Pomel	AY591263
Digitalis cariensis Boiss. ex Jaub. & Spach	AY591282
Digitalis ciliata Trautv	AY591264

**Taxon**	**nrITS**
Digitalis davisiana Heywood	AY591267
Digitalis ferruginea L.	AY591279
Digitalis grandiflora Mill.	AY591261
Digitalis laevigata Waldst. & Kit.	AY591281
Digitalis lanata Ehrh.	AY591285
Digitalis lutea L.	AY591266
Digitalis mariana Boiss.	AY591259
Digitalis minor L.	AY591255
Digitalis nervosa Steud. & Hochst. ex Benth.	AY591283
Digitalis obscura L.	AY591273
Digitalis parviflora Jacq.	AY591286
Digitalis purpurea L.	AY591257
Digitalis subalpina Braun-Blanq.	AY591275
Digitalis thapsi L.	AY591256
Digitalis viridiflora Lindl.	AY591262
Globularia repens Lam.	AY492105
Isoplexis canariensis (L.) Loudon	AY591271
Isoplexis canariensis (L.) Loudon	AF313033
Isoplexis canariensis f. *trichomana*	AY591272
Isoplexis chalcantha Svent. & O'Shan.	AY591269
Isoplexis isabelliana (Webb & Berthel.) Mansf.	AY591270
Isoplexis sceptrum (L. f.) Steud.	AY591268
Plantago atrata Hoppe	AY101895
Plantago coronopus L.	AY101882
Plantago debilis Nees	AY101868
Plantago major L.	AY101861
Plantago raoulii Decne.	AY101867
Plantago spathulata Hook.f.	AY101869
Plantago stauntonii Reichardt	AY101870
Plantago uniflora L.	AY101885
Veronica filiformis Sm.	GU143559

Taxon sampling *Heberdenia excelsa* and *Pleiomeris canariensis* (Primulaceae)

**Taxon**	**nrITS**
Anagallis arvensis L.	AF547739
Androsace brevis (Hegetschw.) Ces.	AY275049
Androsace chaixii Gren. & Godr.	AY275044
Androsace ciliata DC.	AY275034
Androsace elongata L.	AY275072
Androsace filiformis Retz.	AY275078
Androsace halleri L.	AY275037
Androsace hausmannii Leyb.	AY275055

Taxon	nrITS
Androsace hookeriana Klatt	AY275096
Androsace lactea L.	AY275069
Androsace lactiflora Kar. & Kir.	AY275075
Androsace lanuginosa Wall.	AJ491441; AJ491685
Androsace lehmanniana Spreng.	AY275086
Androsace maxima L.	AY275099
Androsace septentrionalis L.	AY275074
Androsace villosa L.	AY275083
Androsace zambalensis (Petitm.) Hand.-Mazz.	AY275091
Ardisia aberrans (E. Walker) C. T. Wu & C. Chen	JN645198
Ardisia depressa C. B. Clarke	JN645199
Ardisia hanceana Mez	JN645187
Ardisiandra wettsteinii R. Wagner	AJ491426; AJ491670
Asterolinon adoense Kunze	AY855152
Bryocarpum himalaicum Hook. f. & Thomson	AJ491428; AJ491672
Centunculus minimus L.	AJ491422; AJ491666
Clavija costaricana Pittier	HQ284101
Coris monspeliensis L.	AF163998; AF163998
Cortusa matthioli L.	AY566867
Cyclamen hederifolium Aiton	AF164004; AF164004
Deherainia smaragdina Decne	AJ491449; AJ491690
Dionysia aretioides (Lehm.) Boiss.	AY680723
Dodecatheon meadia L.	AF164016; AF164016
Douglasia arctica Hook.	AY275016
Douglasia montana A. Gray	AY275023
Embelia ribes Burm. f.	JQ436583
Embelia tsjeriam-cottam (Roem. & Schult.) A. DC.	JQ436584
Glaux maritima L.	AF547728
Heberdenia excelsa (Aiton) Banks	*KJ189028*
Heberdenia excelsa (Aiton) Banks	*KJ189029*
Heberdenia excelsa (Aiton) Banks	*KJ189030*
Heberdenia excelsa (Aiton) Banks	*KJ189031*
Heberdenia excelsa (Aiton) Banks	*KJ189032*
Hottonia palustris L.	AJ491430; AJ491674
Lysimachia nummularia L.	FJ529522
Lysimachia thyrsiflora L.	AF547725
Maesa indica (Roxb.) A. DC.	JQ436585
Maesa japonica (Thunb.) Moritzi & Zoll.	JF708192
Myrsine africana L.	AF164018
Myrsine divaricata A. Cunn.	EF660534
Myrsine faberi (Mez) Pipoly & C. Chen	AF547731
Myrsine oliveri Allan	DQ499109
Myrsine salicina Heward	EU169119
Omphalogramma vinciflorum Franch.	JF977211
Pelletiera wildpretii Valdés	AJ491415; AJ491659

**Taxon**	**nrITS**
Pleiomeris canariensis (Willd.) DC.	*KJ189026*
Pleiomeris canariensis (Willd.) DC.	*KJ189027*
Pomatosace filicula Maxim.	AY275077
Primula forrestii Balf. f.	AJ491438; AJ491682
Primula kitaibeliana Schott	AY275015
Primula palinuri Petagna	AF260758
Primula veris L.	AF323701
Primula verticillata Forssk.	HM629103
Rapanea howittiana F. Muell. ex Mez	DQ499108
Samolus valerandi L.	HQ284122
Soldanella montana Willd.	AF164015; AF164015
Stimpsonia chamaedryoides Wright	AJ491425; AJ491669
Trientalis europaea L.	AF547727
Vitaliana primuliflora Bertol.	AY275050

Taxon sampling *Rhamnus glandulosa* (Rhamnaceae)

**Taxon**	**nrITS**
Berchemia discolor (Klotzsch) Hemsl.	AY626455
Colubrina asiatica (L.) Brongn.	AF328831
Colubrina reclinata (L'Hér) Brongn.	AF328832
Condalia microphylla Cav.	AY626456
Frangula alnus Mill.	AY626431
Frangula baetica (Willk. & E. Rev.) Grubov	AY626450
Frangula betulifolia (Greene) Grubov	AY6264451
Frangula caroliniana (Walter) A. Gray	AY626444
Frangula crenata (Siebold & Zucc.) Miq.	AY626443
Rhamnella franguloides (Maxim.) Weberb.	AY626454
Rhamnidium elaeocarpum Reissek	AY626452
Rhamnus alaternus L.	*KJ188993*
Rhamnus alaternus L.	*KJ188994*
Rhamnus alaternus L.	*KJ188995*
Rhamnus alaternus L.	*KJ188996*
Rhamnus alaternus L.	*KJ188997*
Rhamnus alaternus L.	*KJ188998*
Rhamnus alpina L.	AY626438
Rhamnus californica Eschsch.	AY626442
Rhamnus crenulata Aiton	AY626448
Rhamnus crenulata Aiton	*KJ188990*
Rhamnus crenulata Aiton	*KJ188991*
Rhamnus crenulata Aiton	*KJ188992*
Rhamnus crenulata Aiton	*KJ188999*

Taxon	nrITS
Rhamnus crocea Nutt.	AY626434
Rhamnus davurica Pall.	AY626441
Rhamnus esquirolii H. Lév.	AY626440
Rhamnus glandulosa Aiton	AY626446
Rhamnus glandulosa Aiton	*KJ188986*
Rhamnus glandulosa Aiton	*KJ188987*
Rhamnus glandulosa Aiton	*KJ188988*
Rhamnus glandulosa Aiton	*KJ188989*
Rhamnus glandulosa Aiton	*KJ189000*
Rhamnus glandulosa Aiton	*KJ189001*
Rhamnus glandulosa Aiton	*KJ189002*
Rhamnus glandulosa Aiton	*KJ189003*
Rhamnus glandulosa Aiton	*KJ189004*
Rhamnus integrifolia DC.	*KJ188985*
Rhamnus lycioides subsp. *oleoides* (L.) Jahand. & Maire	*KJ189005*
Rhamnus prinoides L'Hér.	AY626432
Rhamnus pumila Turra	AY626433
Rhamnus purpurea Edgew.	AY626439
Rhamnus purshiana DC.	AY626430
Rhamnus saxatilis Jacq.	AY626447
Rhamnus staddo A. Rich.	AY626449
Sageretia thea (Osbeck) M. C. Johnst.	AY626453
Schistocarpaea johnsonii F. Muell.	AY911539
Scutia buxifolia Reissek	AY626451

Taxon sampling *Prunus lusitanica* ssp. *hixa* and *Prunus lusitanica* ssp. *azorica* (Rosaceae)

Taxon	nrITS
Holodiscus discolor (Pursh) Maxim.	EU669091
Physocarpus opulifolius (L.) Maxim.	JQ034169
Prunus africana (Hook.f.) Kalkman	EU669109
Prunus arborea (Blume) Kalkman	JQ776895
Prunus buergeriana Miq.	JQ926628
Prunus grayana Maxim.	JQ776857
Prunus himalayana (Hook. f. & Thomson) J. Wen	HM856814
Prunus hypoleuca (Koehne) J. Wen	JQ776888
Prunus incisoserrata (T.T. Yu & T.C. Ku) J. Wen	JQ776889
Prunus integrifolia Walp.	HM856811
Prunus javanica (Teijsm. & Binn.) Miq.	JQ776839
Prunus laurocerasus L.	FJ899100
Prunus laurocerasus L.	*KM458055*
Prunus lusitanica L.	JQ776840
Prunus lusitanica L. ssp. *azorica* (Mouillef.)Franco	KC862356

Taxon	nrITS
Prunus lusitanica L. ssp. *azorica* (Mouillef.)Franco	KC862357
Prunus lusitanica L. ssp. *azorica* (Mouillef.)Franco	KC862358
Prunus lusitanica L. ssp. *azorica* (Mouillef.)Franco	KC862359
Prunus lusitanica L. ssp. *azorica* (Mouillef.)Franco	KC862360
Prunus lusitanica L. ssp. *azorica* (Mouillef.)Franco	KC862361
Prunus lusitanica L. ssp. *azorica* (Mouillef.)Franco	KC862362
Prunus lusitanica L. ssp. *azorica* (Mouillef.)Franco	KC862363
Prunus lusitanica L. ssp. *azorica* (Mouillef.)Franco	KC862364
Prunus lusitanica L. ssp. *azorica* (Mouillef.)Franco	KC862365
Prunus lusitanica L. ssp. *azorica* (Mouillef.)Franco	KC862366
Prunus lusitanica L. ssp. *azorica* (Mouillef.)Franco	KC862367
Prunus lusitanica L. ssp. *azorica* (Mouillef.)Franco	KC862368
Prunus lusitanica L. ssp. *azorica* (Mouillef.)Franco	KC862369
Prunus lusitanica L. ssp. *azorica* (Mouillef.)Franco	KC862370
Prunus lusitanica L. ssp. *azorica* (Mouillef.)Franco	KC862371
Prunus lusitanica L. ssp. *hixa* (Willd.)Franco	*KM458056*
Prunus lusitanica L. ssp. *hixa* (Willd.)Franco	*KM458052*
Prunus lusitanica L. ssp. *hixa* (Willd.)Franco	KC862377
Prunus lusitanica L. ssp. *hixa* (Willd.)Franco	KC862378
Prunus lusitanica L. ssp. *hixa* (Willd.)Franco	KC862379
Prunus lusitanica L. ssp. *hixa* (Willd.)Franco	KC862380
Prunus lusitanica L. ssp. *hixa* (Willd.)Franco	*KM458054*
Prunus lusitanica ssp. *lusitanica* L.	*KM458053*
Prunus lusitanica ssp. *lusitanica* L.	KC862372
Prunus lusitanica ssp. *lusitanica* L.	KC862373
Prunus lusitanica ssp. *lusitanica* L.	KC862374
Prunus lusitanica ssp. *lusitanica* L.	KC862375
Prunus lusitanica ssp. *lusitanica* L.	KC862376
Prunus maackii Rupr.	JQ034152
Prunus mahaleb L.	AF318747
Prunus minutiflora Engelm. ex A.Gray	JQ926608
Prunus padus L.	JQ926626
Prunus persica (L.) Batsch	DQ006273
Prunus phaeosticta (Hance) Maxim.	EU669095
Prunus serotina Ehrh.	DQ006035
Prunus spinulosa Siebold & Zucc.	JQ776844
Prunus virginiana L.	JQ926625
Prunus wallichii Steud.	HM856806
Prunus wilsonii (C.K.Schneid.) Koehne	JQ034162

Taxon sampling *Bystropogon* sect. *Canariense*

Taxon	nrITS
Bystropogon canariensis (L.) L′ Hèr.	AY706475

Bystropogon canariensis (L.) L′ Hèr.	AY704592
Bystropogon canariensis (L.) L′ Hèr.	-
Bystropogon canariensis (L.) L′ Hèr.	AY04590
Bystropogon canariensis (L.) L′ Hèr.	AY704593
Bystropogon canariensis (L.) L′ Hèr.	AY04583
Bystropogon canariensis (L.) L′ Hèr.	AY506634
Bystropogon maderensis Webb	AY506633
Bystropogon odoratissimus C. Bolle	AY704589
Bystropogon origanifolius L′ Hèr. 1	AY704585
Bystropogon origanifolius L′ Hèr.	AY704588
Bystropogon origanifolius L′ Hèr.	AY704587
Bystropogon origanifolius L′ Hèr.	AY704591
Bystropogon origanifolius L′ Hèr.	AY506635
Bystropogon plumosus (L. f.) L′ Hèr.	AY704586
Bystropogon punctatus L′ Hèr.	AY704582
Bystropogon wildpretii I. La-Serna	AY704584
Mentha suaveolens Ehrh.	AY506645
Minthostachys mollis (Kunth) Griseb.	AY506638
Pycnanthemum incanum (L.) Michx.	AY506640
Ziziphora hispanica L.	AF369162
