# Supplementary material for: Biogeography of Mediterranean Hotspot Biodiversity: Re-Evaluating the 'Tertiary Relict' Hypothesis of Macaronesian Laurel Forests
Source: PLoS One. 2015 Jul 14;10(7):e0132091. doi: 10.1371/journal.pone.0132091 (PMC4501571; doi:10.1371/journal.pone.0132091)
Supplement: S7 Table — Coded regions: E: Europe/Tethys, M: Macaronesia, S: Asia, A: North & South America, F: Africa; Rel. Prob.: relative probability. Estimates above 10% are marked bold. (PDF) [file pone.0132091.s007.pdf]

S7 Table. Results of the Ancestral area reconstruction using Lagrange. Coded regions: E: Europe/Tethys, M: Macaronesia, S: Asia, A: North & South America, F: Africa; Rel.Prob.: relative probability. Estimates above 10% are marked bold.

**Taxon**	**E**	**M**	**M-E**	**A**	**M-S**	**S**	**M-S-F**	**M-A**	**E-A**	**F**	**E-S**	**M-F**	**F-S**	**S-A**	**M-E-S**	**Sum of Rel. Prob**
Aeonium cuneatum		**100**														100.0
Aichryson pachycaulon agg.		**98**														98.4
Apollonias barbujana				2	2	**79**								**11**		95.0
Arbutus canariensis				**13**				**43**	**41**							97.2
Bystropogon sect. *Canariense*		**100**														100.0
Euphorbia mellifera/ stygiana	**50**		**48**													98.1
Heberdenia excelsa		9			**10**	**46**	**18**					7	7			98.0
Isoplexis group	**100**															100.0
Ixanthus viscosus	**70**	5	**18**	1					0		0				1	94.2
Laurus novocanariensis/ azorica	**75**		**18**						4							96.7
Persea indica		6		**85**				5								96.2
Picconia excelsa/ azorica	**34**	**38**	**19**	1	7	4				1		0				103.9
Pleiomeris canariensis		9			**46**		**35**					7				97.6
Prunus lusitanica	**26**				**21**	**27**					**21**					96.0
Rhamnus glandulosa	**59**		**40**													99.2
Sambucus nigra subsp. palmensis	**55**	2	**39**													96.6
Viburnum rigidum		4	**92**													96.9
**percentage [%]**	28.0	22.2	15.8	6.1	5.2	9.3	3.2	2.8	2.7	0.0	1.3	0.8	0.4	2.0	0.1	
